# Engineered RebH Halogenase Variants Demonstrating a Specificity Switch from Tryptophan towards Novel Indole Compounds

**DOI:** 10.1002/cbic.202100210

**Published:** 2021-07-29

**Authors:** Barindra Sana, Timothy Ho, Srinivasaraghavan Kannan, Ding Ke, Eunice H. Y. Li, Jayasree Seayad, Chandra S. Verma, Hung A. Duong, Farid J. Ghadessy

**Affiliations:** ^1^ Disease Intervention Technology Laboratory Institute of Molecular and Cell Biology Agency for Science Technology and Research (A*STAR) 8 A Biomedical Grove, #06-04/05 Neuros/Immunos Singapore 138648 Singapore; ^2^ Institute of Chemical and Engineering Sciences Agency for Science Technology And Research (A*STAR) 8 Biomedical Grove, Neuros, #07-01 Singapore 138665 Singapore; ^3^ Bioinformatics Institute Agency for Science Technology And Research (A*STAR) 30 Biopolis Street, #07-01 Matrix Singapore 138671 Singapore; ^4^ School of Biological Sciences Nanyang Technological University 60 Nanyang Drive Singapore 637551 Singapore; ^5^ Department of Biological Sciences National University of Singapore 14 Science Drive 4 Singapore 117558 Singapore

**Keywords:** biocatalysis, directed evolution, enzyme engineering, halogenase, indole, RebH

## Abstract

Activating industrially important aromatic hydrocarbons by installing halogen atoms is extremely important in organic synthesis and often improves the pharmacological properties of drug molecules. To this end, tryptophan halogenase enzymes are potentially valuable tools for regioselective halogenation of arenes, including various industrially important indole derivatives and similar scaffolds. Although endogenous enzymes show reasonable substrate scope towards indole compounds, their efficacy can often be improved by engineering. Using a structure‐guided semi‐rational mutagenesis approach, we have developed two RebH variants with expanded biocatalytic repertoires that can efficiently halogenate several novel indole substrates and produce important pharmaceutical intermediates. Interestingly, the engineered enzymes are completely inactive towards their natural substrate tryptophan in spite of their high tolerance to various functional groups in the indole ring. Computational modelling and molecular dynamics simulations provide mechanistic insights into the role of gatekeeper residues in the substrate binding site and the dramatic switch in substrate specificity when these are mutated.

## Graphical abstract

**Two engineered halogenase enzymes** were developed by switching substrate specificity of the parental enzyme from tryptophan to indole compounds. They can halogenate several industrially important indole derivatives more efficiently than the parental enzyme; this includes enzymatic chlorination/bromination of novel substrates that were not reported previously. The engineered enzymes could be useful in regioselective synthesis of drug intermediates.

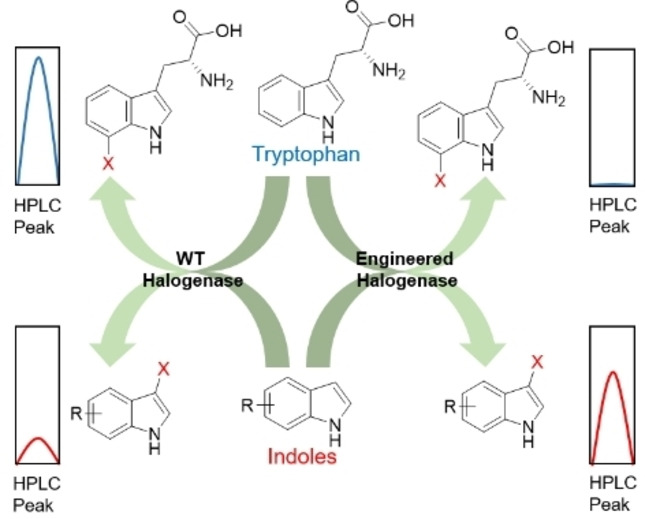

## Introduction

Halogenated aromatic hydrocarbons are very important in the chemical, agrochemical and pharmaceutical industries, not only as active molecules in finished products but also as intermediates in organic synthesis.^[1]^ Halogenation can drastically affect the molecular pharmacology of many drugs that include anticancer, antimicrobial and psychoactive agents.^[2]^ Both the type and the position of halogen substituent can significantly alter metabolism, pharmacokinetics and tissue distribution of drug molecules.^[3]^ Aryl and heteroaryl halides are also important as key intermediates in transition metal‐catalyzed cross‐coupling reactions, which are amongst the most important technologies in synthetic chemistry.

Selective halogenation of arene compounds in mild conditions is exceptionally challenging by conventional chemical synthesis. Chemical halogenation processes often face sustainability issues due to requirement of activated starting materials and multistep synthesis involving hazardous reagents and harsh reaction conditions. In addition, the ability to control the regioselectivity of halogenation remains a fundamental challenge. The lack of selectivity often leads to production of undesirable byproducts including toxic polyhalogenated compounds, which may create challenges in separation^[4]^ and disposal due to human health hazards and environmental persistence. Enzyme‐catalyzed halogenation offers very high specificity and regioselectivity, and the overall synthesis can be performed in fewer steps under milder reaction conditions using simple halide salts as the halogenating reagents, thereby minimizing the generation of harmful waste products.[^4^, ^5^] Flavin‐dependent halogenases (FDH) comprise a group of enzymes that catalyze regioselective halogenation of electron‐rich aromatic compounds including tryptophan and indoles. They play a major role in halogenation of organic molecules during biosynthesis of various natural products including vancomycin, rebeccamycin, chloramphenicol and cryptophycin.^[6]^ FDH‐catalyzed *in vitro* halogenation reactions take place under ecofriendly conditions using inert salts as halogen source and oxygen as the terminal oxidant.[^4^, ^7^]

The indole motif is ubiquitous in bioactive natural products and is a privileged scaffold in drug discovery.^[1c]^ The preparation of functionalized indoles is thus of enormous interest.^[8]^ C−H halogenation of indole and its derivatives is of particular interest since these can be readily transformed into various complex structures.^[9]^ Chemical halogenation of indoles generally occurs on the pyrrole ring via an electrophilic aromatic substitution mechanism. In contrast, FDHs can halogenate various indole compounds including tryptophan and tryptamine.^[10]^ These enzymes have typically evolved to work on specific substrates, and many indoles of potential interest are not efficiently halogenated. Enzyme engineering approaches have been employed to broaden the substrate scope of these enzymes, facilitating direct installation of functional groups at selective positions of various indole compounds that would be impossible otherwise.^[11]^

As chlorides are the most abundant halides on earth and dominate in halogenated natural products,^[12]^ most enzymatic halogenation studies reported to date focus on chlorination. However, enzymatic synthesis of 3‐bromoindoles, which are important intermediates for the preparation of pharmaceuticals and biologically active compounds, is also highly desirable. Furthermore, aryl bromides are more reactive than aryl chlorides in transition metal‐catalyzed cross‐coupling reactions.^[13]^ Several novel FDHs including BrvH, Xcc, SpH1, SpH2 and VirX1 have recently been described that preferentially brominate or iodinate various substrates including diverse indole compounds.[^5a^, ^10b^, ^14^] BrvH, SpH1 and SpH2 showed high conversion rate towards several indolic substrates for production of 3‐bromoindoles. These can potentially be developed for industrial applications. A further validated approach is to use rational or combinatorial engineering to expand the substrate scope of well‐characterized FDHs such as RebH, a tryptophan 7‐halogenase reported from the rebeccamycin biosynthetic pathway.[^6d^, ^15^] This enzyme has been extensively engineered for multiple purposes, including halogenating indole substrates.[^11a^, ^11b^, ^16^] Here, we report improved halogenation of a panel of indole compounds using two RebH variants developed by switching the substrate specificity of RebH through a semi‐rational mutagenesis and screening approach.

## Results and Discussion

### Screening

**Figure 1:**
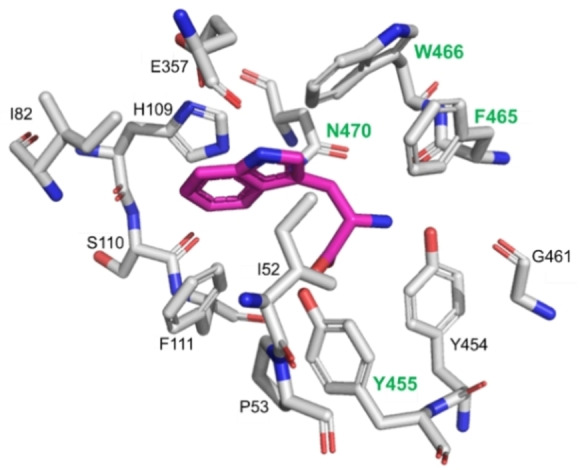
Amino acid residues selected for mutation in the RebH substrate binding site. The structure is adapted from the RebH 3‐LSR structure (PDB code 4LU6^[16d]^) with L‐tryptophan (shown in magenta) superimposed from the structure of wild‐type RebH bound to L‐tryptophan (PDB code 2E4G^[17]^).

**Figure 2:**
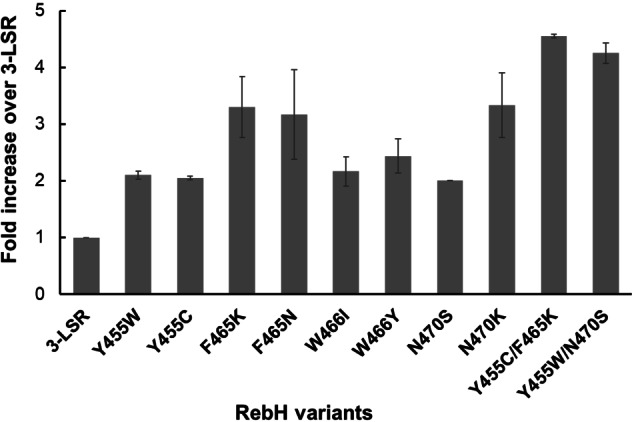
Selected enzyme variants show increased indole 6‐carboxylic acid bromination activity. Activity of the indicated point mutants is represented as fold increase over the parental 3‐LSR enzyme. n=3±SD.

Given that thermostability is a key consideration in enzymatic process development, we used the previously reported thermostable RebH 3‐LSR variant (hereafter termed 3‐LSR) as the parental enzyme to make focused libraries.^[16d]^ Residues within a 4 Å radius of the putative tryptophan binding site of 3‐LSR (Figure 1) were individually mutated to generate 13 focused libraries. Screening of bacterial lysates (∼2000 clones) for bromination of indole‐6 carboxylic acid yielded 8 variants with≥2‐fold higher activity over parental 3‐LSR: Y455W, Y455C, F465K, F465N, W466I, W466Y, N470S and N470K (Figure 2). Notably, these mutated residues cluster in the binding pocket region that predominantly interacts with the tryptophan carbonyl and amide backbone (Figure 1).

Beneficial point mutations were next combined to generate 15 double mutant enzyme variants (Figure S1 in the Supporting Information). These were purified and tested for bromination of indole 6‐caroboxylic acid. Two variants, Y455C/F465K and Y455W/N470S (hereafter referred to as M1 and M2 respectively) showed 4.5‐ and 4.2‐fold improved conversion respectively, outperforming the best singleton variants (Figure 2, Figure S1 in the Supporting Information). Most combinations of the mutations did not show any additive affects. In particular, 80 % activity loss was observed for the F465K/N470K and F465N/N470K double mutants (Figure S1 in the Supporting Information). Close proximity of these residues in the structure may account for this observation (Figure 1). The mutations in M1 have not been previously described. Both the Y455W and N470S mutations in M2 have been previously reported, either in isolation or in combination with other mutations but not exclusively together.[^11a^, ^11b^, ^16a^]

### Substrate scope

**Figure 3:**
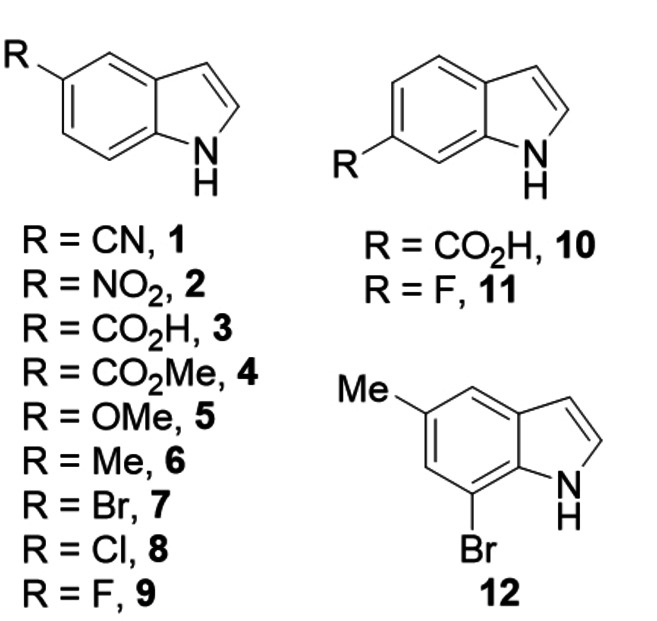
The indole compounds tested for halogenation.

**Figure 4:**
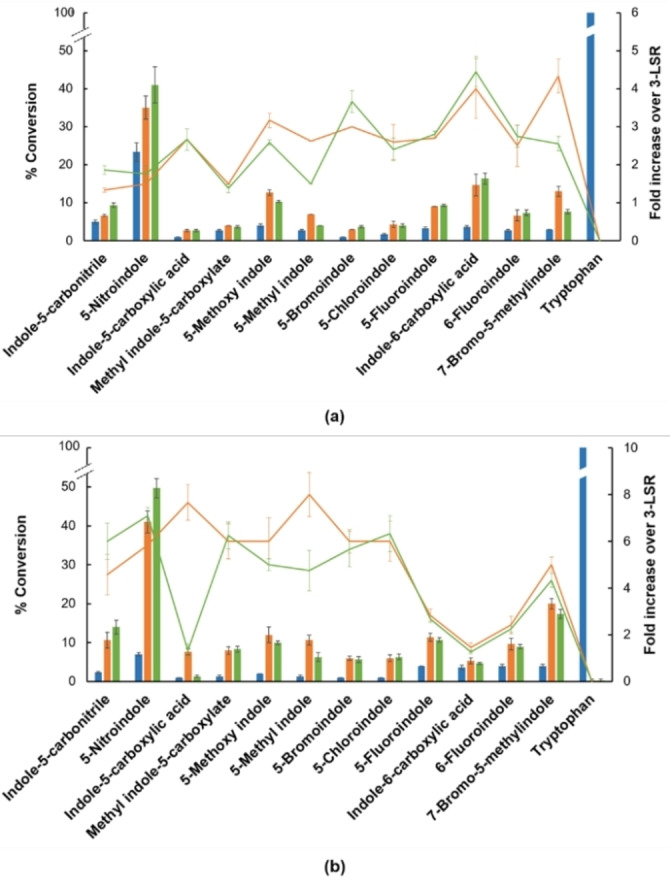
RebH 3‐LSR variants display improved activity on novel indole substrates. a) Bromination of indole substrates with RebH 3‐LSR (blue), M1 (green) and M2 (orange) mutants. Conversion (%) of each substrate is shown as a bar chart and the improvement of activity compared to RebH 3‐LSR variant is shown as a line. n=3±SD. b) Same as in a) but for chlorination reactions.

Activities of the M1 and M2 variants were next evaluated for both chlorination and bromination of an extended panel of indole compounds to understand substrate scope and relative efficacy compared to the parental 3‐LSR enzyme (Figure 3). Although selected for improved bromination of indole 6‐carboxylic acid, the two variants showed similarly improved activity over 3‐LSR for all the indole derivatives tested (Figure 4). Both exhibited >2‐fold increased bromination of indole‐5‐carboxylic acid (**3**), 5‐bromoindole (**7**), 5‐chloroindole (**8**), 5‐fluoroindole (**9**), indole‐6 carboxylic acid (**10**), 6‐fluoroindole (**11**) and 7‐bromo‐5‐methylindole (**12**). Chlorination efficiencies were likewise generally improved across the panel with both enzymes showing >2‐fold increased activity with 5‐nitroindole (**2**), methyl‐indole‐5‐carboxylate (**4**), 5 methoxy indole (**5**), 5‐methyl indole (**6**), 5‐bromoindole (**7**), 5‐chloroindole (**8**), 5‐fluoroindole (**9**) and 7‐bromo‐5‐methylindole (**12**). Notably, whilst M2 showed ∼8‐fold improved bromination of indole‐5‐carboxylic acid, activity of M1 was the same as 3‐LSR. Despite bromination being selected for during enzyme screening the mutants were still largely more proficient at chlorination, which could be related to the inherent function of RebH in the biosynthesis of chlorinated natural products. An exception was indole‐6‐carboxylic acid, with bromination by both enzyme variants ∼2‐fold more efficient than chlorination. The highest conversion was achieved for the 5‐nitroindole (**2**) substrate using the M1 mutant, achieving 50 % chlorination and 40 % bromination at completion after overnight incubation. Corresponding values for 3‐LSR were 23 % and 7 % respectively. By way of comparison, 99 %, 89 %, 52 % and 97 % bromination of **1**, **2**, **7** and **9**, has been reported using BrvH.^[10b]^ SpH1 and SpH2 also showed efficient bromination of substrates **2**, **7** and **9**.^[14c]^

The selected variants M1 and M2 were completely inactive towards the natural substrate tryptophan for both chlorination and bromination reactions. The same phenotype was observed with lower tryptophan concentrations, ruling out substrate inhibition (Table S2 in the Supporting Information). Although mutation at the active site of FDHs is known to generate enzymes with dramatically altered substrate specificity, a complete switch of substrate activity is relatively rare.[^11b^, ^16a^] This switch likely arises through position 455 as the Y455W mutation has previously been shown to reduce activity towards tryptophan with concomitant increased turnover of tryptamine.^[16a]^ The F465K and N470S mutations in M1 and M2 respectively further reduce tryptophan specificity to undetectable levels. Furthermore, both Y455W and N470S mutations in M2 are also present along with 8 other mutations in a RebH variant selected for altered regioselectivity on tryptamine that is inactive towards tryptophan.^[11a]^ The N470S mutation in M2 has also been previously described to increase activity and/or alter regioselectivity towards non‐natural substrates.^[11b]^ Interestingly, the N470S variation prevails in several endogenous bacterial tryptophan‐7 halogenases whilst the Y455 and F465 residues are highly conserved (Figure S2 in the Supporting Information). Circular dichroism (CD) analysis indicated no major changes in secondary structures of these mutants compared to 3‐LSR (Figure S3 in the Supporting information).

NMR spectroscopy was next used to determine selectivity of the two variants and parental enzyme on the 5‐nitroindole substrate. All three enzymes produced only 3‐chloro‐5‐nitroindole or 3‐bromo‐5‐nitroindole (Figures S4–S7 in the Supporting Information), which is particularly interesting considering their use as precursors for synthesis of antitumor agents and HDAC inhibitors, respectively.^[18]^ This is the first report of enzymatic synthesis of 3‐chloro‐5‐nitroindole. Similarly, halogenated indole‐5‐carbonitriles are useful in the synthesis of LSD1 inhibitors,^[19]^ and for the first time we report enzymatic chlorination of this compound. Although 3‐LSR was able to halogenate both 5‐nitroindole (**2**) and the indole‐5‐carbonitrile (**1**), conversions were significantly improved by using the engineered enzymes (Figure 4). The brominated product of **9**, 3‐bromo‐5‐fluoro indole has been reported in several patents for its application in synthesis of various pharmaceutical compounds including antithrombotic agents, CRTh2 antagonists and lactate dehydrogenase inhibitors. Brominated products of compounds **3**, **5**, **8** and **10**, and chlorinated products of compounds **3**, **5** and **10** have also been reported as pharmaceutical intermediates in several patents. Halogenations were done using hazardous reagents including molecular halogen or *N*‐halosuccinimide in selective organic solvents such as dimethylformamide, chloroform and pyridine. In contrast, the enzymatic halogenations reported here were carried out in aqueous media at room temperature using benign salts as halogenating agents.

### Predicting enzyme‐substrate interactions

**Figure 5:**
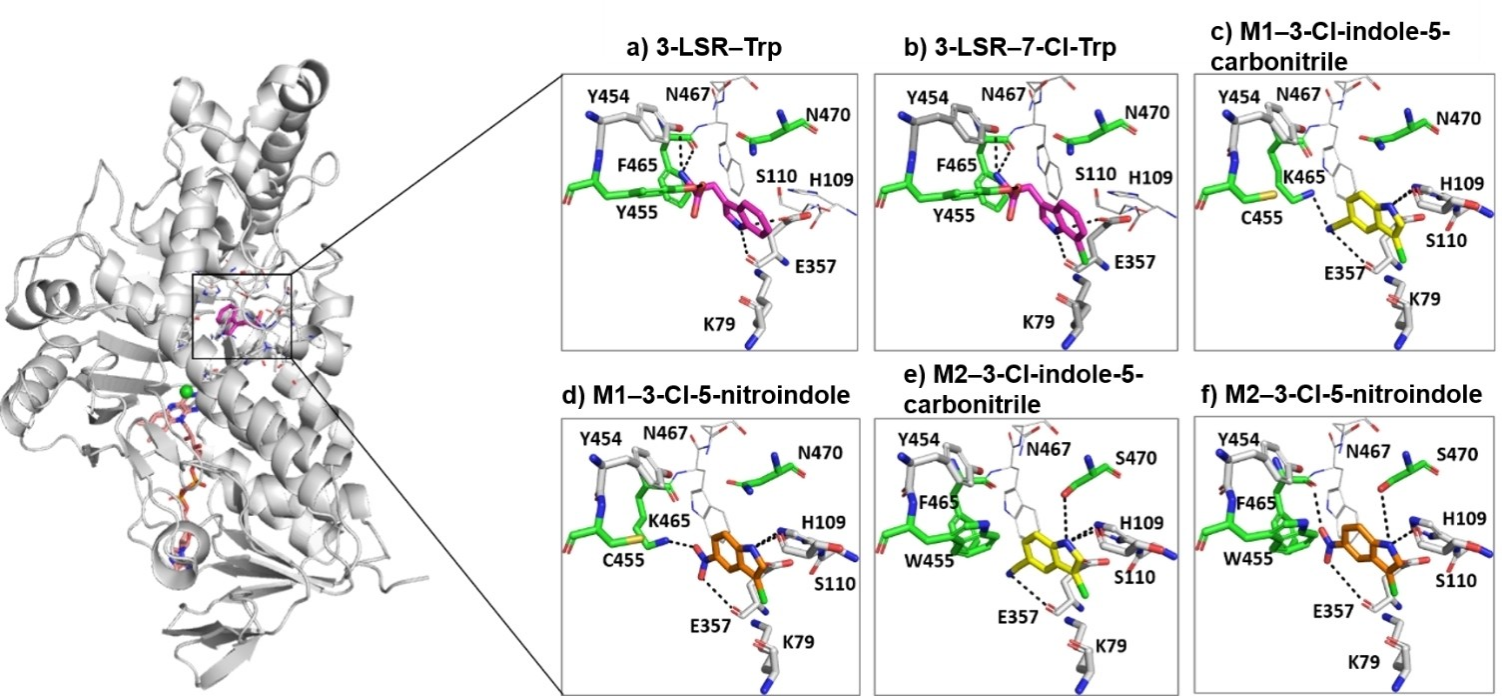
Left: Overall structure of the 3‐LSR – FAD/Cl/Trp complex. 3‐LSR is shown as a grey cartoon and the bound FAD (orange sticks), Cl (green sphere), Trp (magenta sticks) are highlighted. Right: Close‐up view of snapshots of 3‐LSR and M1 (Y455C/F465K) and M2 (Y455W/N470S) enzymes bound to Trp and indole derivatives. For clarity only the active site residues that are interacting with the ligands are shown in sticks (grey). The residues that are mutated in M1 and M2 are in green and the bound ligands are colored separately. The protein‐ligand interactions are highlighted in dashed lines (black).

In the co‐crystal structure of the RebH‐Trp complex, the Trp is bound via extensive hydrogen bonding mostly involving its carbonyl and amide backbone and packing interactions. The carbonyl backbone of Trp interacts with the side chain of Y455 whilst its amide backbone interacts with the side chains of residues Y454 and E461, and with the carbonyl backbone of residue F465 from RebH. The side chain nitrogen of Trp interacts with the carbonyl backbone of residue E357 from RebH. In addition, the binding of Trp is involved in packing interactions with His109 (Figure 5a). These interactions are crucial for the RebH enzyme to orient the substrate to control halogenation selectivity. Molecular dynamics (MD) simulations have been useful in delineating halogenase‐substrate interactions.[^11a^, ^20^] MD simulations initiated from either the experimental structure or the docked complex showed a stable bound conformation of Trp, and interactions with residues from the active site of RebH are well preserved. Similarly, the bound conformation of 7‐chloro Trp also remained stable and maintained interactions with the 3‐LSR active site residues (Figure 5b). In contrast, the conformations of Trp and 7‐chloro Trp were not stable during simulations with the M1 and M2 variants. The Trp backbone makes extensive H‐bond interactions with RebH/3‐LSR, particularly with residues Y455 and F465. These are both mutated in M1 (to Cys and Lys respectively), resulting in loss of stabilizing contacts and binding to Trp. In the case of M2, the substitution of Y455 with a bulky Trp results in a steric clash with the substrate backbone, destabilizing the complex during MD simulations (Figure S8 in the Supporting Information). This loss of both stabilizing interactions and steric clashes explains the inefficacy of M1 and M2 towards halogenation of Trp as the conformationally stable binding of Trp is crucial for proper positioning of the indole side chain for efficient halogenation. A similar loss of contacts is predicted for the BrvH and VirX1 enzymes that are inactive towards tryptophan and lack the well‐defined loop comprising Y455 and F465 that covers the active site of RebH (Figure S9 in the Supporting information).[^5a^, ^10b^]

We next carried out simulations to explore the binding of 3‐LSR, M1 and M2 to indole‐5 carbonitrile (**1**), 5‐nitro‐indole (**2**) and their chlorinated analogues. The bound conformations of **1**, **2** and their chlorinated versions derived from the bound conformation of Trp were not stable during the MD simulations of the 3‐LSR, M1 and M2 complexes (Figure S8 in the Supporting Information). Compared to Trp, both **1**, **2** and their chlorinated analogues lack the backbone amide and carbonyl groups, therefore no interactions were observed between the 3‐LSR mutants and the bound ligand (Figure S8 in the Supporting Information). In contrast, the bound conformation of **1**, **2** and their chlorinated analogues obtained from the docking calculations remained stable during the MD simulations. The docking of these indole derivatives revealed a conformation in which the indole ring adopted a flipped conformation (Figure 5c–5f). A similar altered conformation was noticed in a previous study where tryptamine was docked to the crystal structure of RebH.^[11a]^ In this conformation the indole nitrogen from **1** interacts with the sidechain of residue H109 and backbone carbonyl of S110 of M1. In addition, the carbonitrile interacts with the side chain of K465 and backbone carbonyl of E357 from M1. The 5‐carbon position of the indole derivative is pointing towards the side chain of K79 and is within 4.5 Å from the side chain nitrogen (Figure 5c). Similar interaction patterns were also observed for chlorinated **1**; the Cl is within 3 Å from the side chain nitrogen of K79. A similar bound pose was also observed for **2** and its chlorinated analogue (Figure 5d). Instead of the carbonitrile, the nitro group interacts with the side chain of K465 and with the backbone carbonyl of E357 from M1. The 5‐carbon position of the indole derivative for the non‐chlorinated analogue and the Cl of the chlorinated analogue points towards the side chain of K79 and is within 4.5 Å and 3 Å respectively from the side chain nitrogen.

The binding poses of M2 with **1**, **2** and their chlorinated analogues were very similar to those of M1 (Figure 5e and 5f). The bound indole derivatives are involved in H‐bond interactions with the side chain of H109 and with the backbone carbonyl of S110. The carbonitrile interacts with the backbone carbonyl of E357, whereas the nitro group interacts with the backbone carbonyl of E357 and F465. In addition, the nitrogen atom from indole derivatives interacts with the side chain oxygen of S470. Notably, the structurally equivalent residue in BrvH is also serine (S447), and the same H‐bond interaction likely contributes to its reported activity on **1** and **2** (Figure S9 in the Supporting information).^[10b]^ The bound conformation of the indole derivatives is further stabilized by packing interactions involving residues H109 and W455 from M2. As in M1, the 5‐carbon position/Cl of the indole derivatives both point towards the side chain of K79 and are again within 4.5 Å and 3 Å respectively from the side chain nitrogen. The Y455W (M1) and N470S (M2) mutations in RebH have previously been identified as determinants of substrate specificity, both increasing preference for tryptamine over tryptophan as the preferred substrate and improving chlorination on non‐native substrates.[^11a^, ^11b^, ^16a^] Our results further confirm their roles in directing substrate specificity.

We next looked at whether the improved halogenation activities of M1 and M2 on the other indole substrates studied here could be explained by the predicted binding mode of **1** and **2**. Most of the indole derivatives (except the indole‐6‐carboxylic acid (**10**) and 6‐fluroindole (**11**)) have substitutions at position 5, similar to **1** and **2**. Likewise, these substitutions can engage in either H‐bond interactions (carboxylic acid substitutions, carboxylate and methoxy) or halogen bond interactions (bromo, chloro and fluoro). Although the methyl group at position 5 in the case of 5‐methyl cannot be involved in H‐bond interactions, it can participate in tight packing with proximal active site hydrophobic residues. Therefore, the 5‐subsitituted indole derivatives can bind stably to M1 and M2 with concomitant halogenation at position 3. The 6‐substituted indole derivatives can also bind similarly, however in the case of the 6‐caroxylic acid substituted indole, the interaction with the backbone carbonyl of E357 will be replaced by H‐bond interactions with Y454, K465, N467 and N470 of M1 and Y454, N467 and S470 of M2. The fluorine in the 6‐fluoro substituted indole forms halogen bond interactions with the side chain of N470 and with the backbone carbonyl of K465 in M1 and interacts with the backbone carbonyl of F465 in M2. The 7‐bromo‐5‐methylindole (**12**) can also bind similarly, with the bromine at position 7 engaging in halogen bond interaction with N470 in M1 and S470 in M2.

## Conclusion

We have expanded the catalytic scope of the RebH enzyme by switching substrate specificity through a semi‐rational enzyme engineering approach. Two RebH variants have been identified that are completely inactive on tryptophan but show higher efficiency for halogenating a broad spectrum of indoles. Enzymatic halogenation of many of these indoles has not been previously described. Several important pharmaceutical precursors were also synthesized by site‐selective halogenation of various indole compounds using the engineered enzymes. In addition to their use in halogenating novel substrates for synthetic chemistry, these enzymes with altered substrate selectivity can potentially be exploited as a metabolic engineering tool for biosynthesis of novel natural products.

## Experimental Section

**Cloning and library preparation**: Given that thermostability is a key consideration in enzymatic process development, we used the previously reported thermostable RebH 3‐LSR variant (hereafter termed 3‐LSR) as the parental enzyme to make focused libraries.^[16d]^ The gene encoding 3‐LSR was codon‐optimized for recombinant expression in *E. coli*, and amplified by PCR using 5’‐AAGGA GATAT ACATA TGCCA GGTAA AATAG ATAAA ATTCT TATTG TTGGA GGAGG GA‐3’ and 5’‐GGTGG TGGTG CTCGA GTCTA CCATG TTGCT GTCTA AGAAA TTCAT GT‐3’ primers. The amplified fragment was gel‐purified and inserted in NdeI/XhoI double‐digested linear pET22b vector by infusion cloning. The construct was transformed into chemically competent DH5α cells, and the sequence of the construct was confirmed by DNA sequencing.

We identified 13 amino acid residues within 4 Å radius of the substrate binding site for making a series of focused libraries (Figure 1). Thirteen individual libraries were made by randomizing the I52, P53, I82, H109, S110, F111, E357, Y454, Y455, G461, F465, W466 and N470 residues separately, so that each position could be mutated to all possible 20 natural amino acids without inserting any stop codons. Randomization was carried out by PCR following a previously reported method.^[21]^ The PCR was done using a specific ratio of three primers containing NDT, VHG and TGG codons, and their reverse complements at the randomization sites (primer sequences are listed in Table S1 in the Supporting Information). 10 μM of each primer solution was made in water. Three forward primers and three reverse primers were mixed separately at molar ratio of 12 : 9 : 1 prior to use for PCR. PCR was done in a 50 μL reaction mixture containing 50 ng template, 5 μL Pfu reaction buffer, 5 μL 2 mM dNTP mix, 3 μL DMSO, 2.5 μL Forward primer mix, 2.5 μL of Reverse primer mix and 1 μL Pfu Turbo enzyme. The mutant genes in the PCR products were amplified, inserted into NdeI/XhoI double‐digested pET22b vector and transformed in DH5α cells as described above. Sequences of the constructs and diversity in individual libraries was confirmed by sequencing 16 clones from each library. Colonies from each library were pooled together separately and grown overnight prior to plasmid isolation.

**Screening**: Screening was carried out for bromination of indole‐6‐carboxylic acid. Plasmids from each library were transformed into chemically competent BL21(DE3) cells. Screening was done in 24‐well plates. Individual clones were grown overnight in LB media containing 50 μg ml^−1^ kanamycin. The overnight cultures were diluted 1 : 100 in Terrific Broth (TB) media containing 50 μg ml^−1^ kanamycin and grown at 37 °C for 3 hours followed by 25 °C for overnight. Cells were collected by centrifugation and lysed using B‐PER II bacterial protein extraction reagent (ThermoFisher Scientific). The supernatants were collected and used as source of enzyme in the enzyme activity assay for bromination of indole‐6‐carboxylic acid. 180 μL supernatant was used to brominate 2.5 mM indole‐6‐carboxylic acid in the presence of 50 mM NaBr and cofactors 10 μM FAD, 2 mM NADH and 30 μM flavin reductase enzyme RebF, 20 mM glucose and 5 units glucose dehydrogenase enzyme in phosphate buffer pH 7.2. Reactions were incubated overnight at room temperature with constant mixing, and were stopped by heating at 95 °C for 5 minutes. Precipitates were removed by centrifugation at 13,500 rpm for 10 minutes and the supernatants were analyzed by HPLC. The conversions were calculated from the Area Under Curve and compared with that of the parental 3‐LSR enzyme. Clones with higher activity were sequenced to confirm mutations. Individual mutants were over‐expressed at medium scale and the proteins were purified. The activities of purified mutant enzymes were measured for enzymatic bromination of indole‐6‐carboxylic acid and compared with that of the 3‐LSR enzyme. Variants with ≥2x activity compared to 3‐LSR enzyme were selected for further mutagenesis through combination of beneficial mutations to make double‐mutants. Activities of purified double mutant enzymes were measured for bromination of indole‐6‐carboxylic acid, and compared with that of the single mutants and the 3‐LSR variant.

**Enzyme expression and purification**: Plasmid constructs were transformed into *E. coli* BL21(DE3) competent cells following manufacturer's protocol (NEB) and grown overnight on LB‐agar plates containing 50 μg ml^−1^ kanamycin at 37 °C. Overnight cultures were prepared by inoculating single colonies in LB broth containing 50 μg ml^−1^ kanamycin and grown at 37 °C with constant shaking. For overexpression, the overnight cultures were diluted 100‐fold in the same media, grown at 37 °C until the OD600 reached to 0.5, and induced with 0.5 mM IPTG. The cells were harvested after culturing overnight at 25 °C, and stored at −80 °C.

The cells were lysed by sonication and the proteins were purified using Ni‐NTA column (GE healthcare) following standard protocols. The purified proteins were concentrated and buffer exchanged into 50 mM phosphate buffer pH 7.5 using 10 kDa MWCO Amicon Ultra centrifugal filters. Concentration of the purified proteins was determined by OD280 using Nanodrop and their purity was analyzed by SDS‐PAGE.

**Enzyme activity measurement**: Enzymatic activity of the purified 3‐LSR variants was measured on tryptophan and various indole compounds (Figure 3). 2.5 mM substrate was treated with 10 μM enzyme and 50 mM NaCl/NaBr in the presence of the cofactors 10 μM FAD, 2 mM NADH and 30 μM flavin reductase enzyme RebF, 20 mM glucose and 5 units glucose dehydrogenase enzyme. The reaction was done in 10 mM phosphate buffer (pH=7.2), overnight at room temperature with constant mixing. The enzymes were inactivated by heating at 95 °C for 5 minutes and removed by centrifugation at 13,500 rpm for 10 minutes. The supernatant was analyzed by HPLC, and the corresponding halogenated product was quantified from the Area Under Curve.

**Halogenation position of indole compounds**: 5‐Nitroindole was chlorinated and brominated at large scale using 3‐LSR and the two most active mutant enzymes. After overnight reactions, the enzymes were precipitated by heating and removed by centrifugation. The supernatants were lyophilized and the resultant residues were dissolved in methanol followed by purification of the halogenated products by semi‐preparative HPLC. The halogenation site was identified by ^1^H NMR spectroscopy.

**Analytical methods**: Chemicals and anhydrous solvents were obtained from Sigma Aldrich and were used without further purification. Spectroscopic grade solvents were purchased from Sigma Aldrich. NMR spectra were recorded on Bruker Avance III 400 MHz spectrometer in MeOD‐d4. Data are reported in the following order: chemical shifts are given (δ); multiplicities are indicated as s (singlet), d (doublet), t (triplet), q (quartet) and m (multiplet). High‐resolution mass spectra (HRMS) were recorded on an Agilent ESI‐TOF mass spectrometer at 3500 V emitter voltage. Exact m/z values are reported in Daltons.

For analytical HPLC, 20 μL of crude mixture was injected onto an Agilent EclipsePlus C18 analytical column (1.8 μ packing, 2.1 mm×50 mm). Gradient starting conditions of 10 % (v/v) MeCN/H_2_O (plus 0.1 % (v/v) HCOOH) were held for 1 min before development to 95 % (v/v) MeCN/H_2_O over 3 min prior to re‐equilibration to starting conditions over 2 min. Flow rates and column temperature were kept constant at 0.4 mL min^−1^ and 25 °C respectively. UV absorbance was detected at 254 nm and 280 nm throughout.

For semi‐preparative HPLC, 900 μL of solution containing crude mixture dissolved in H_2_O/MeCN was injected onto a Phenomenex Jupiter semi‐preparative C12 HPLC column (4 μ packing, 250×10 mm). Starting conditions of 50 % (v/v) MeCN/H_2_O (plus 0.05 % (v/v) TFA) were held for 2 min. prior to development to 90 % (v/v) MeCN/H_2_O over 15 min. 95 % (v/v) MeCN/H_2_O then held for 3 min. prior to re‐equilibration of starting conditions over 3 min. Flow rates were kept constant at 5 ml min^−1^. UV absorbance was detected at 280 nm throughout.

**In‐silico modelling and MD simulations**: The crystal structure of the enzyme RebH in its apo (PDB: 2OAM^[17]^), FAD & Cl bound (PDB: 2OAL^[17]^) and Trp bound forms (PDB: 2E4G^[17]^) and the 3‐LSR in its apo form (PDB: 4LU6^[16d]^) are available. 3‐LSR is structurally very similar to the RebH – FAD/Cl and Trp complexes with rmsd <0.5 Å. Using these structures, a structural model of 3‐LSR – FAD/Cl/Trp complex was generated. Using this modelled structure of the complex, the structures of selected 3‐LSR double mutants (Y455W/N470S and Y455C/F465K) in their apo form and in complex with FAD/Cl/Trp were generated. Two sets of structural models of 3‐LSR and its mutants with the chlorinated and non‐chlorinated Trp, indole derivatives (5‐nitro‐indole, indole‐5 carbonitrile) were generated. In one set the binding pose of the molecules were derived from the conformation of Trp bound with RebH and in the other set, the binding poses were generated using in‐silico docking (explained below).

The 3D structures of Trp, 5‐nitro‐indole (**2**), indole‐5‐carbonitrile (**1**) and their chlorinated versions (7‐chloro‐Trp, 3‐chloro‐5‐nitro‐indole, 3‐chloro‐indole‐5‐carbonitrile) were built using *Maestro* and prepared with the Ligprep module employing the OPLS‐2005 force field in Schrodinger 12.0.^[22]^ The prepared ligands were docked into the active site of the chosen structure using *Glide*.^[23]^ A box of size 10×10×10 Å centered on the selected active site residues (the active site was defined as the region where the Trp was bound in the co‐crystal structure of the RebH – Trp complex) was used to restrict the search space of each docked ligand. Default *Glide* settings were used to generate the grids. The docking protocol was first validated by redocking the Trp into the active site of RebH that resulted in the docked pose of Trp to be very similar (rmsd 0.3 Å) to the experimentally observed conformation of Trp. Docking was carried out using rigid RebH variants and flexible ligands. The docked conformation of each ligand was evaluated using the *Glide* Extra Precision (XP) scoring function. Docking was carried out with 3‐LSR and its mutant structures. The top scoring binding pose was selected for further refinement.

In both the modelled and docked structures of 3‐LSR and its mutants, ligand molecules were subject to refinement using MD simulations. The simulations were carried out using the *pmemd.CUDA* module of the program Amber18.^[24]^ The partial charges and force field parameters for the cofactors and the ligands were generated using the *Antechamber* module in Amber18. All atom versions of the Amber 14SB force field (ff14SB)^[25]^ and the general Amber force field (GAFF)^[26]^ were used to model the protein and the cofactors/ligands respectively. The *Xleap* module was used to prepare the system for the MD simulations. Each simulation system was neutralized with an appropriate number of counterions. Each neutralized system was solvated in an octahedral box with TIP3P^[27]^ water molecules, with at least a 10 Å boundary between the solute atoms and the borders of the box. During the simulations, LJ and short‐range electrostatic interactions were treated using a cut‐off scheme and the long‐range electrostatic interactions were treated with the particle mesh Ewald method^[28]^ using a real space cut‐off distance of 9 Å. The Settle^[29]^ algorithm was used to constrain bond vibrations involving hydrogen atoms, which allowed a time step of 2 fs during the simulations. Solvent molecules and counterions were initially relaxed using energy minimization with restraints on the protein and inhibitor atoms. This was followed by unrestrained energy minimization to remove any steric clashes. Subsequently the system was gradually heated from 0 to 300 K using MD simulations with positional restraints (force constant: 50 kcal mol^−1^ Å^−2^) on the protein and cofactors/ligands over a period of 0.25 ns allowing water molecules and ions to move freely. During an additional 0.25 ns, the positional restraints were gradually reduced followed by a 2 ns unrestrained MD simulation to equilibrate all the atoms. For each system, a 250 ns production MD run at 300 K in triplicate (assigning different initial velocities to propagate each MD simulation) was carried out. Simulation trajectories were visualized using VMD^[30]^ and figures were generated using PyMOL.^[31]^

## Conflict of interest

The authors declare no conflict of interest.

## Supporting information

As a service to our authors and readers, this journal provides supporting information supplied by the authors. Such materials are peer reviewed and may be re‐organized for online delivery, but are not copy‐edited or typeset. Technical support issues arising from supporting information (other than missing files) should be addressed to the authors.

Supporting Information
