# Supplementary material for: Engineered RebH Halogenase Variants Demonstrating a Specificity Switch from Tryptophan towards Novel Indole Compounds
Source: Chembiochem. 2021 Jul 29;22(18):2791–8. doi: 10.1002/cbic.202100210 (PMC8518859; doi:10.1002/cbic.202100210)
Supplement: Supplementary file 1 — Supporting Information [file CBIC-22-2791-s001.pdf]

ChemBioChem

Supporting Information

Engineered RebH Halogenase Variants Demonstrating a Specificity Switch from Tryptophan towards Novel Indole Compounds

Barindra Sana, Timothy Ho, Srinivasaraghavan Kannan, Ding Ke, Eunice H. Y. Li, Jayasree Seayad, Chandra S. Verma, Hung A. Duong, and Farid J. Ghadessy*

Table S1. List of oligonucleotides used for making saturation libraries

Target residues	Degenerated codons	Forward Primers	Reverse primers
I52	NDT	5'-AGGCCACA**NDT**CCGAACCTTCAGACAG-3'	5'-CTGTCTGAAGGTTCGG**AHN**TGTGGCCT-3'
	VHG	5'-AGGCCACA**VHG**CCGAACCTTCAGACAG-3'	5'-CTGTCTGAAGGTTCGG**CDB**TGTGGCCT-3'
	TGG	5'-AGGCCACA**TGG**CCGAACCTTCAGACAG-3'	5'-CTGTCTGAAGGTTCGG**CCA**TGTGGCCT-3'
P53	NDT	5'-AGGCCACAATA**NDT**AACCTTCAGACAG-3'	5'-CTGTCTGAAGGTT**AHN**TATTGTGGCCT-3'
	VHG	5'-AGGCCACAATA**VHG**AACCTTCAGACAG-3'	5'-CTGTCTGAAGGTT**CDB**TATTGTGGCCT-3'
	TGG	5'-AGGCCACAATA**TGG**AACCTTCAGACAG-3'	5'-CTGTCTGAAGGTT**CCA**TATTGTGGCCT-3'
I82	NDT	5'-TGCAATGCGAGTTATAAAGTCGCC**NDT**AAATTCATAAATTGGAGAAC-3'	5'-GTTCTCCAATTTATGAATTT**AHN**GGCGACTTTATAACTCGCATTGCA-3'
	VHG	5'-TGCAATGCGAGTTATAAAGTCGCC**VHG**AAATTCATAAATTGGAGAAC-3'	5'-GTTCTCCAATTTATGAATTT**CDB**GGCGACTTTATAACTCGCATTGCA-3'
	TGG	5'-TGCAATGCGAGTTATAAAGTCGCC**TGG**AAATTCATAAATTGGAGAAC-3'	5'-GTTCTCCAATTTATGAATTT**CCA**GGCGACTTTATAACTCGCATTGCA-3'
H109	NDT	5’-AGACCACTTCTAC**NDT**AGCTTCGGTTTACTTAAGTATCACGA-3’	5'-TCGTGATACTTAAGTAAACCGAAGCT**AHN**GTAGAAGTGGTCT-3'
	VHG	5’-AGACCACTTCTAC**VHG**AGCTTCGGTTTACTTAAGTATCACGA-3’	5'-TCGTGATACTTAAGTAAACCGAAGCT**CDB**GTAGAAGTGGTCT-3'
	TGG	5’-AGACCACTTCTAC**TGG**AGCTTCGGTTTACTTAAGTATCACGA-3’	5'-TCGTGATACTTAAGTAAACCGAAGCT**CCA**GTAGAAGTGGTCT-3'
S110	NDT	5’-AGACCACTTCTACCAT**NDT**TTCGGTTTACTTAAGTATCACGA-3’	5'-TCGTGATACTTAAGTAAACCGAA**AHN**ATGGTAGAAGTGGTCT-3'
	VHG	5’-AGACCACTTCTACCAT**VHG**TTCGGTTTACTTAAGTATCACGA-3’	5'-TCGTGATACTTAAGTAAACCGAA**CDB**ATGGTAGAAGTGGTCT-3'
	TGG	5’-AGACCACTTCTACCAT**TGG**TTCGGTTTACTTAAGTATCACGA-3’	5'-TCGTGATACTTAAGTAAACCGAA**CCA**ATGGTAGAAGTGGTCT-3'
F111	NDT	5’-AGACCACTTCTACCATAGC**NDT**GGTTTACTTAAGTATCACGA-3’	5'-TCGTGATACTTAAGTAAACC**AHN**GCTATGGTAGAAGTGGTCT-3'
	VHG	5’-AGACCACTTCTACCATAGC**VHG**GGTTTACTTAAGTATCACGA-3’	5'-TCGTGATACTTAAGTAAACC**CDB**GCTATGGTAGAAGTGGTCT-3'
	TGG	5’-AGACCACTTCTACCATAGC**TGG**GGTTTACTTAAGTATCACGA-3’	5'-TCGTGATACTTAAGTAAACC**CCA**GCTATGGTAGAAGTGGTCT-3'
E357	NDT	5’-TGTGGAACCTCTG**NDT**TCTACAGGAATCTACTTCGT-3’	5'-ACGAAGTAGATTCCTGTAGA**AHN**CAGAGGTTCCACA-3'
	VHG	5’-TGTGGAACCTCTG**VHG**TCTACAGGAATCTACTTCGT-3’	5'-ACGAAGTAGATTCCTGTAGA**CDB**CAGAGGTTCCACA-3'
	TGG	5’-TGTGGAACCTCTG**TGG**TCTACAGGAATCTACTTCGT-3’	5'-ACGAAGTAGATTCCTGTAGA**CCA**CAGAGGTTCCACA-3'

Y454	NDT	8a:5′-GACGATGCGCAACTT**NDT**TATGGAAATTTCGAGGA-3′	5'-TCCTCGAAATTTCCATA**AHN**AAGTTGCGCATCGTC-3'
	VHG	5′-GACGATGCGCAACTT**VHG**TATGGAAATTTCGAGGA-3′	5'-TCCTCGAAATTTCCATA**CDB**AAGTTGCGCATCGTC-3'
	TGG	5′-GACGATGCGCAACTT**TGG**TATGGAAATTTCGAGGA-3′	5'-TCCTCGAAATTTCCATA**CCA**AAGTTGCGCATCGTC-3'
Y455	NDT	9a:5′-GACGATGCGCAACTTTAC**NDT**GGAAATTTCGAGGA-3′	5'-TCCTCGAAATTTCC**AHN**GTAAAGTTGCGCATCGTC-3'
	VHG	5′-GACGATGCGCAACTTTAC**VHG**GGAAATTTCGAGGA-3′	5'-TCCTCGAAATTTCC**CDB**GTAAAGTTGCGCATCGTC-3'
	TGG	5′-GACGATGCGCAACTTTAC**TGG**GGAAATTTCGAGGA-3′	5'-TCCTCGAAATTTCC**CCA**GTAAAGTTGCGCATCGTC-3'
G461	NDT	5′-TCGAGGAA**NDT**TTTCGCAACTTTTGGAATAATTCA-3′	5'-TGAATTATTCCAAAAGTTGCGAAA**AHN**TTCCTCGA-3'
	VHG	5′-TCGAGGAA**VHG**TTTCGCAACTTTTGGAATAATTCA-3′	5'-TGAATTATTCCAAAAGTTGCGAAA**CDB**TTCCTCGA-3'
	TGG	5′-TCGAGGAA**TGG**TTTCGCAACTTTTGGAATAATTCA-3′	5'-TGAATTATTCCAAAAGTTGCGAAA**CCA**TTCCTCGA-3'
F465	NDT	5′-TCGAGGAAGGCTTTCGCAAC**NDT**TGGAATAATTCA-3′	5'-TGAATTATTCCA**AHN**GTTGCGAAAGCCTTCCTCGA-3'
	VHG	5′-TCGAGGAAGGCTTTCGCAAC**VHG**TGGAATAATTCA-3′	5'-TGAATTATTCCA**CDB**GTTGCGAAAGCCTTCCTCGA-3'
	TGG	5′-TCGAGGAAGGCTTTCGCAAC**TGG**TGGAATAATTCA-3′	5'-TGAATTATTCCA**CCA**GTTGCGAAAGCCTTCCTCGA-3'
W466	NDT	5′-TCGAGGAAGGCTTTCGCAACTTT**NDT**AATAATTCA-3′	5'-TGAATTATT**AHN**AAAGTTGCGAAAGCCTTCCTCGA-3'
	VHG	5′-TCGAGGAAGGCTTTCGCAACTTT**VHG**AATAATTCA-3′	5'-TGAATTATT**CDB**AAAGTTGCGAAAGCCTTCCTCGA-3'
	TGG	5′-TCGAGGAAGGCTTTCGCAACTTT**TGG**AATAATTCA-3′	5'-TGAATTATT**CCA**AAAGTTGCGAAAGCCTTCCTCGA-3'
N470	NDT	5′-CTTTTGGAATAATTCA**NDT**TATTACTGCGTCTTAGCTGGTCTG-3′	5'-CAGACCAGCTAAGACGCAGTAATA**AHN**TGAATTATTCCAAAAG-3'
	VHG	5′-CTTTTGGAATAATTCA**VHG**TATTACTGCGTCTTAGCTGGTCTG-3′	5'-CAGACCAGCTAAGACGCAGTAATA**CDB**TGAATTATTCCAAAAG-3'
	TGG	5′-CTTTTGGAATAATTCA**TGG**TATTACTGCGTCTTAGCTGGTCTG-3′	5'-CAGACCAGCTAAGACGCAGTAATA**CCA**TGAATTATTCCAAAAG-3'

Table S2. Conversion (%) of tryptophan at low concentrations using the RebH variants

Tryptophan Concentration	3-LSR		M1		M2	
	Bromination	Chlorination	Bromination	Chlorination	Bromination	Chlorination
10 μM	100 ± 0	100 ± 0	0	0	0	0
50 μM	97 ± 3	100 ± 0	0	0	0	0
100 μM	98 ± 2	100 ± 0	0	0	0	0

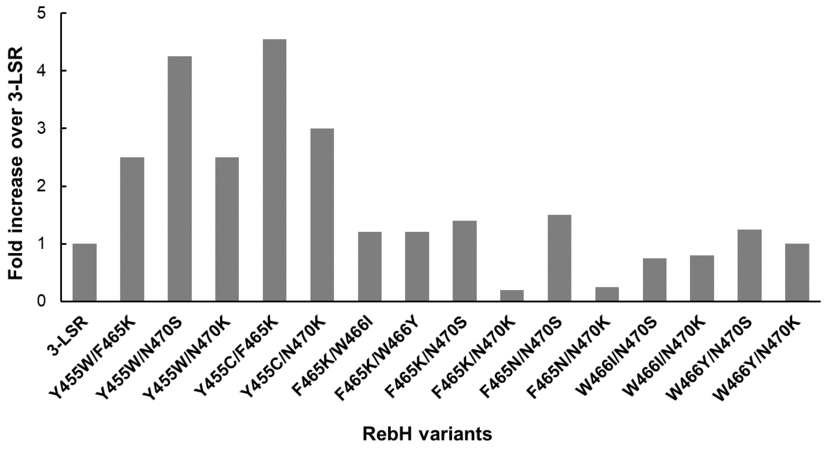

Figure S1. Indole 6-carboxylic acid bromination activity by double mutants of 3-LSR (n = 1). Activity of the indicated variants is presented as fold increase over the parental 3-LSR enzyme.

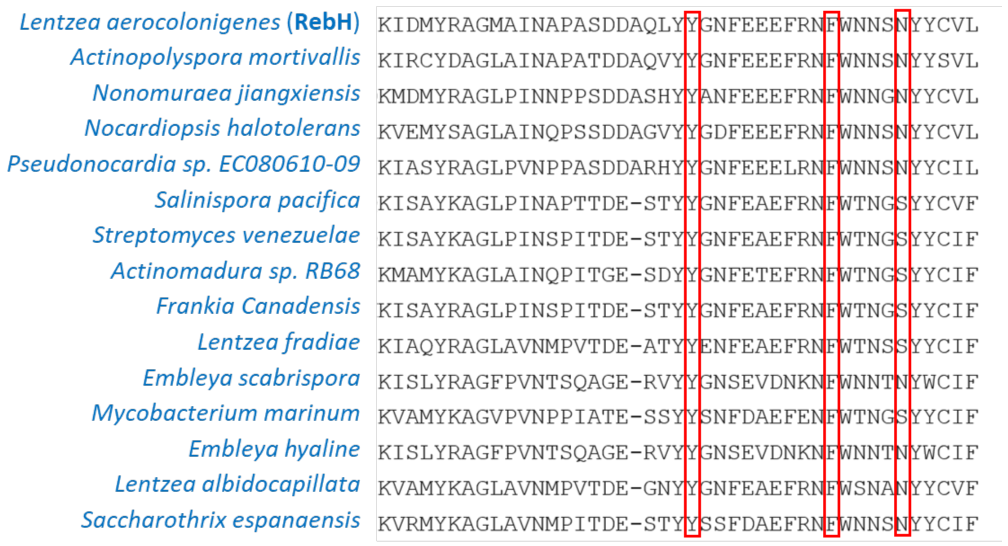

Figure S2. Sequence alignment of RebH with other bacterial tryptophan 7-halogenase enzymes showing the conservation at Y455, F465 and N470 residues.

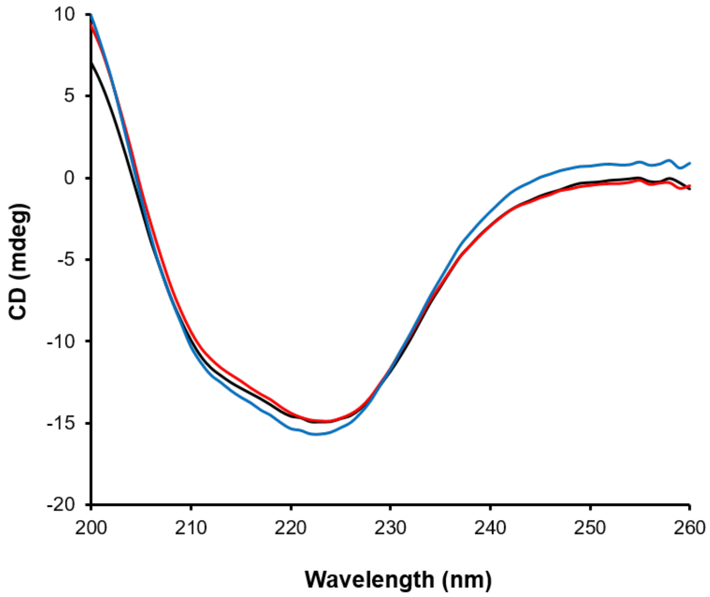

Figure S3. CD spectra of 3-LSR (black), M1 (red) and M2 (blue); the data is average of two independent measurements.

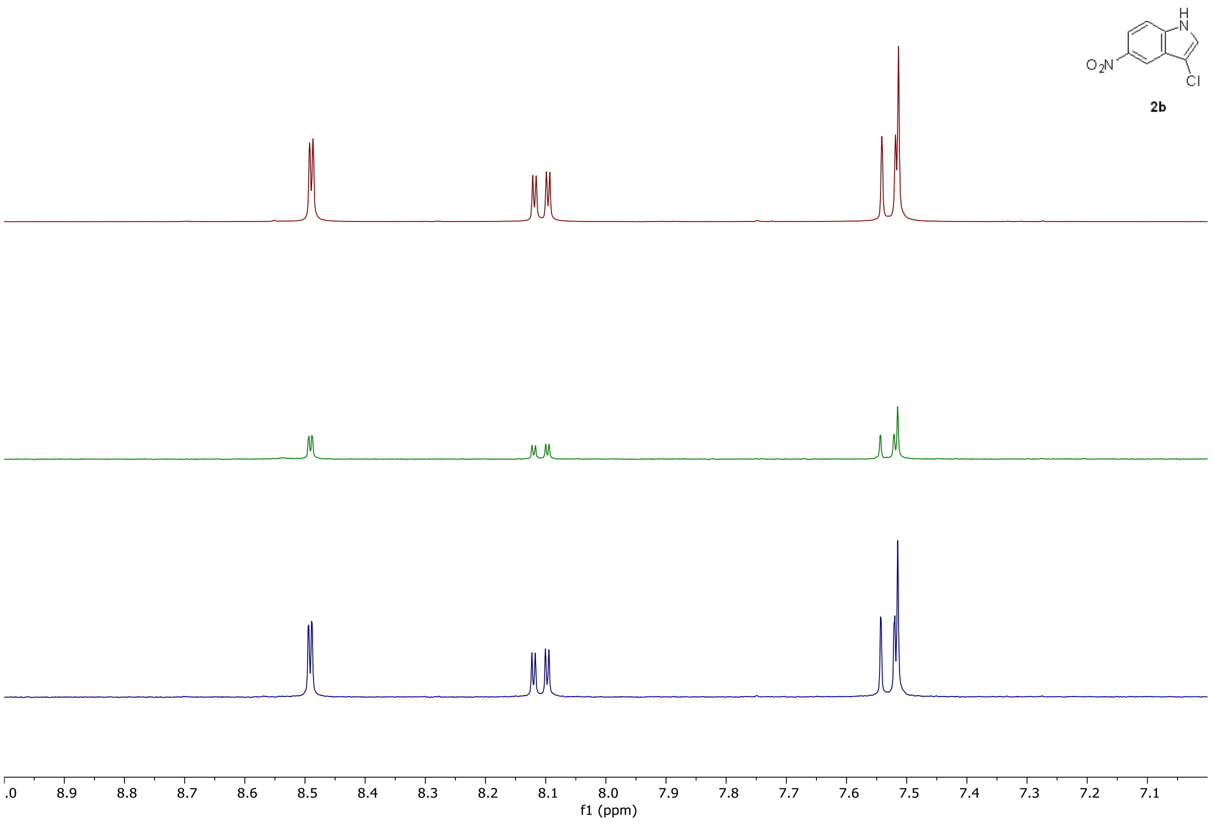

Figure S4. ^{1}H stacked spectra of 3-chloro-5-nitro-1H-indole produced by enzymatic halogenation using (A) M2, (B) M1 and (C) 3-LSR.

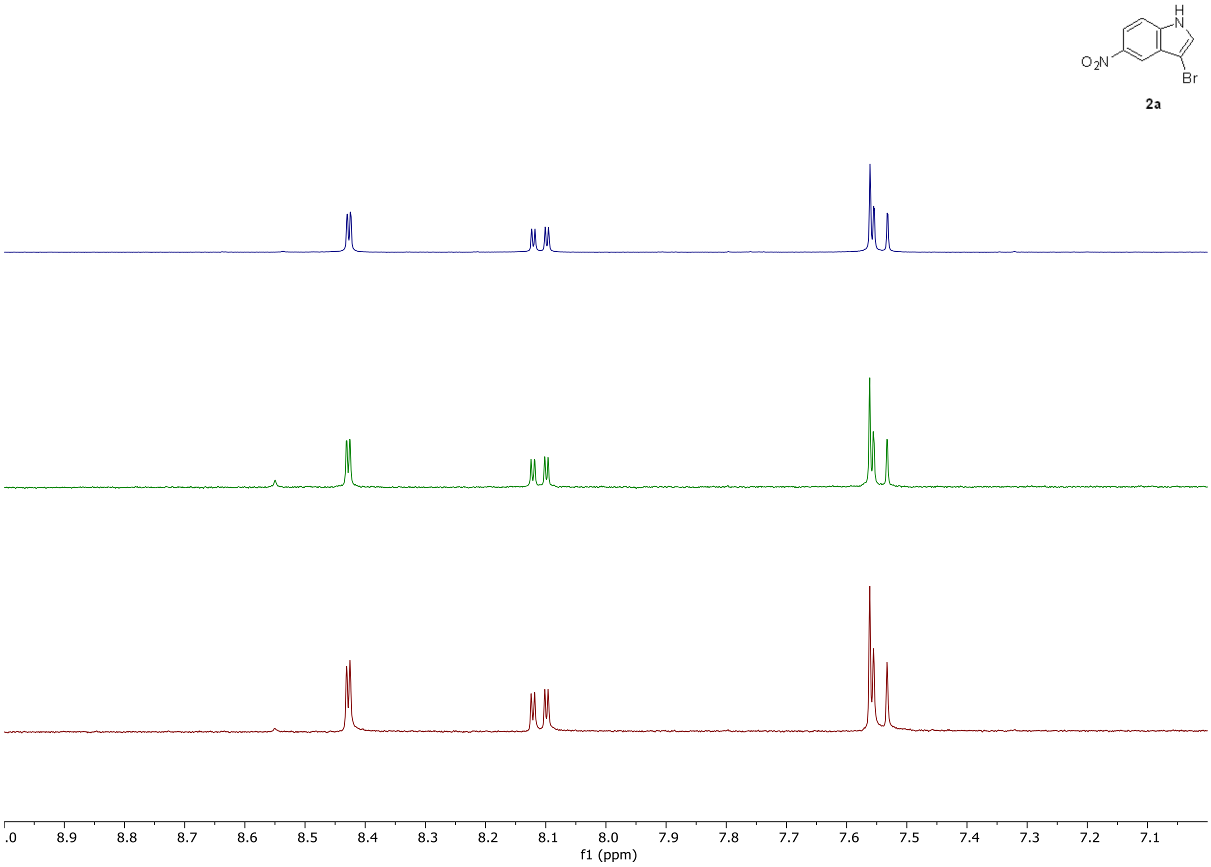

Figure S5. ^1H stacked spectra of 3-bromo-5-nitro-1H-indole produced by enzymatic halogenation using (A) M2, (B) M1 and (C) 3-LSR.

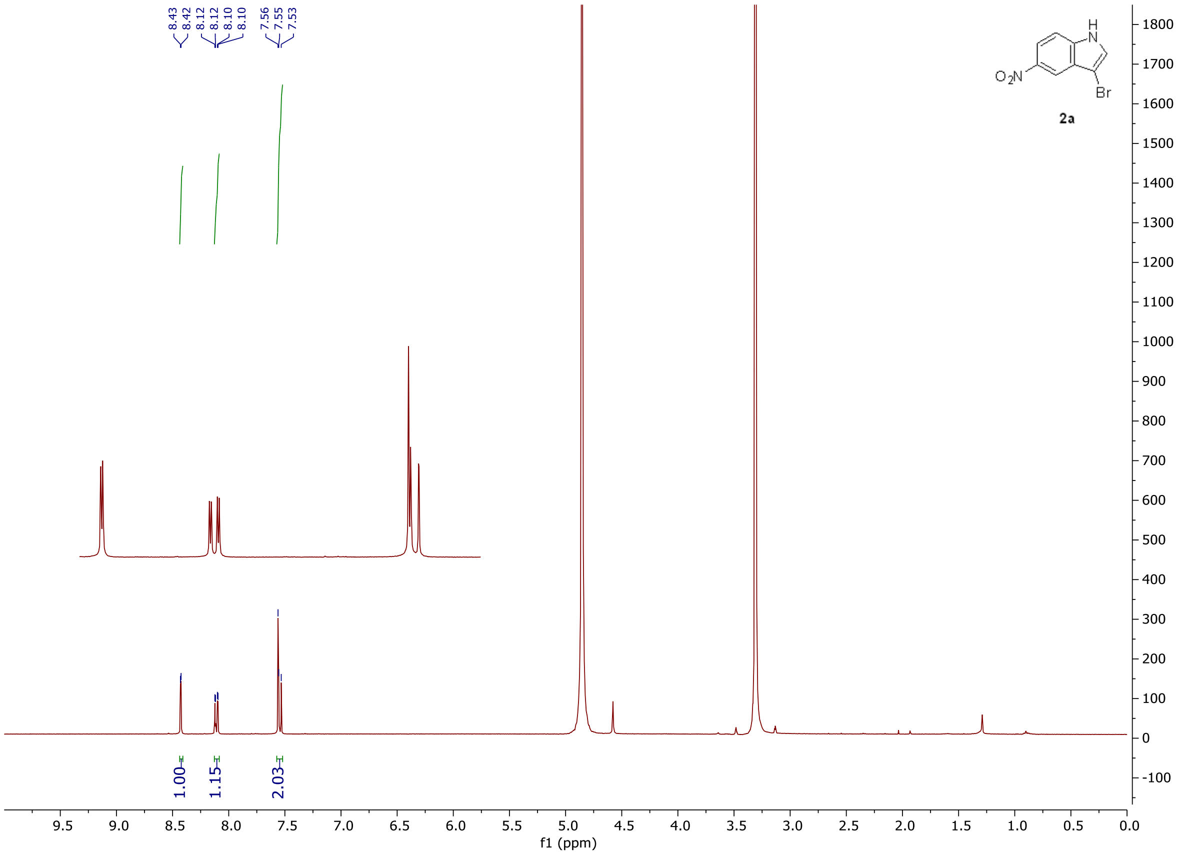

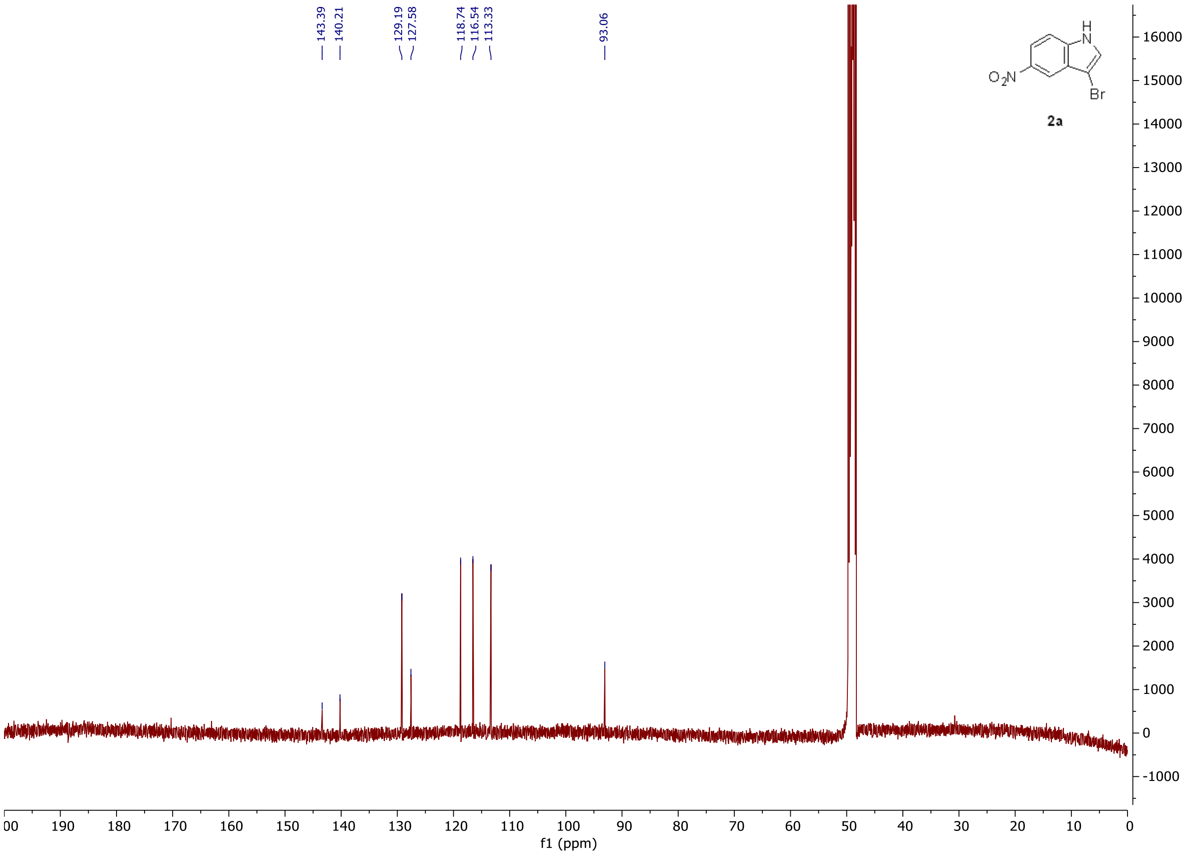

Figure S6. ^{1}H and ^{13}C spectra of 3-bromo-5-nitro-1H-indole.

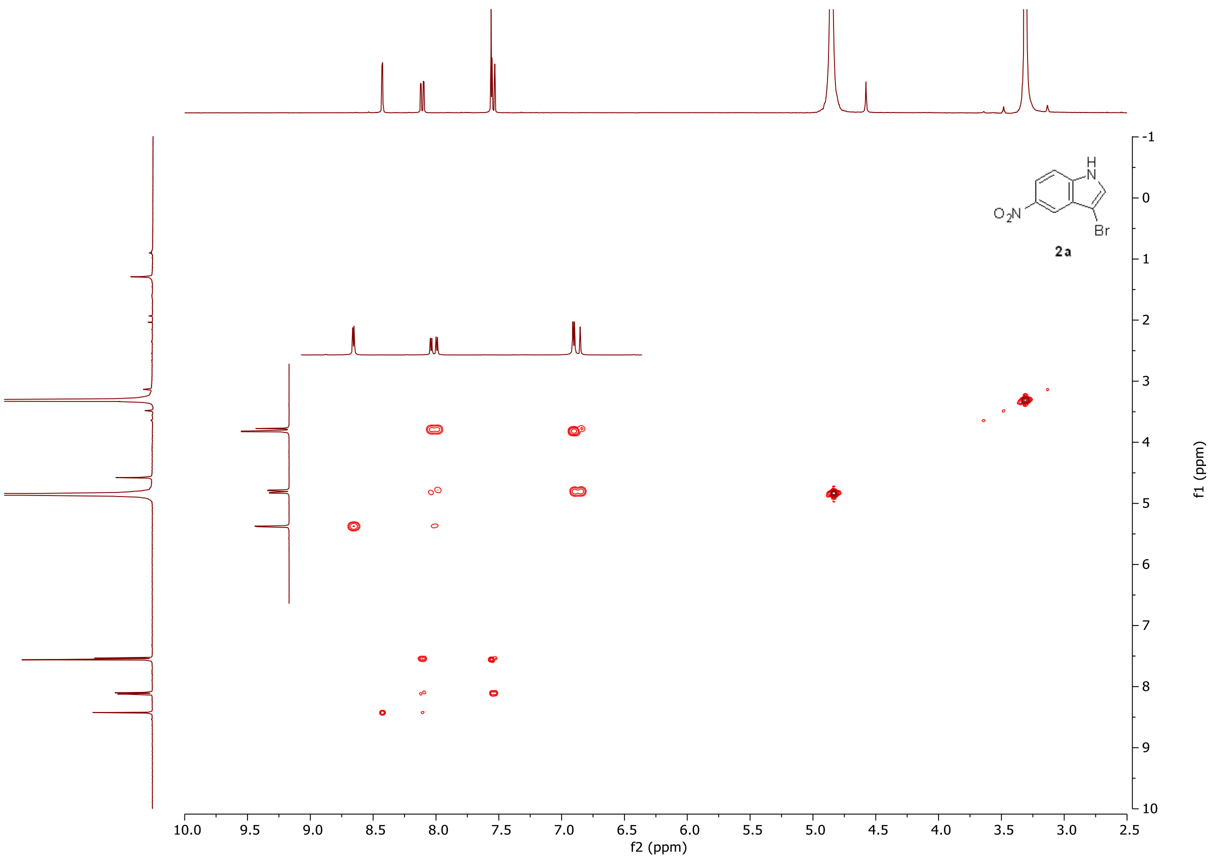

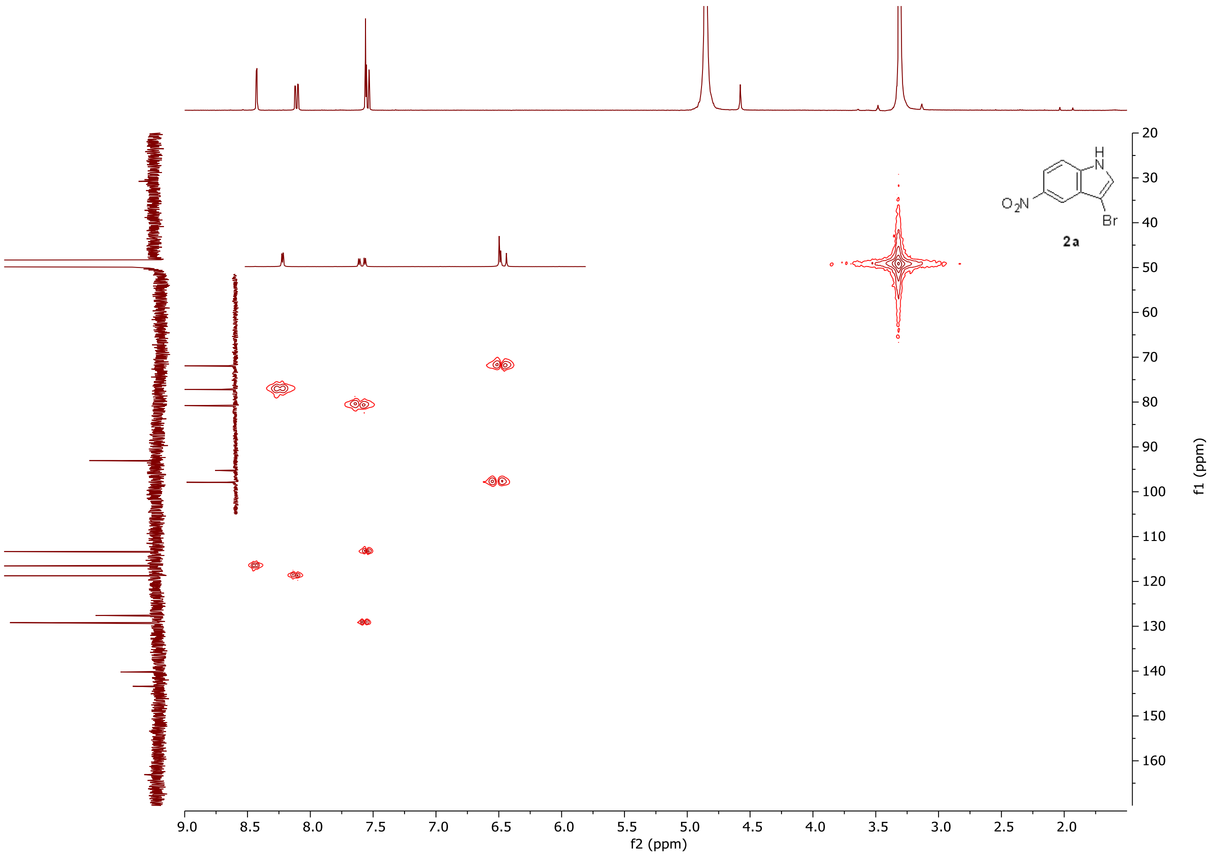

Figure S7. COSY and HMQC spectrum of 3-bromo-5-nitro-1H-indole.

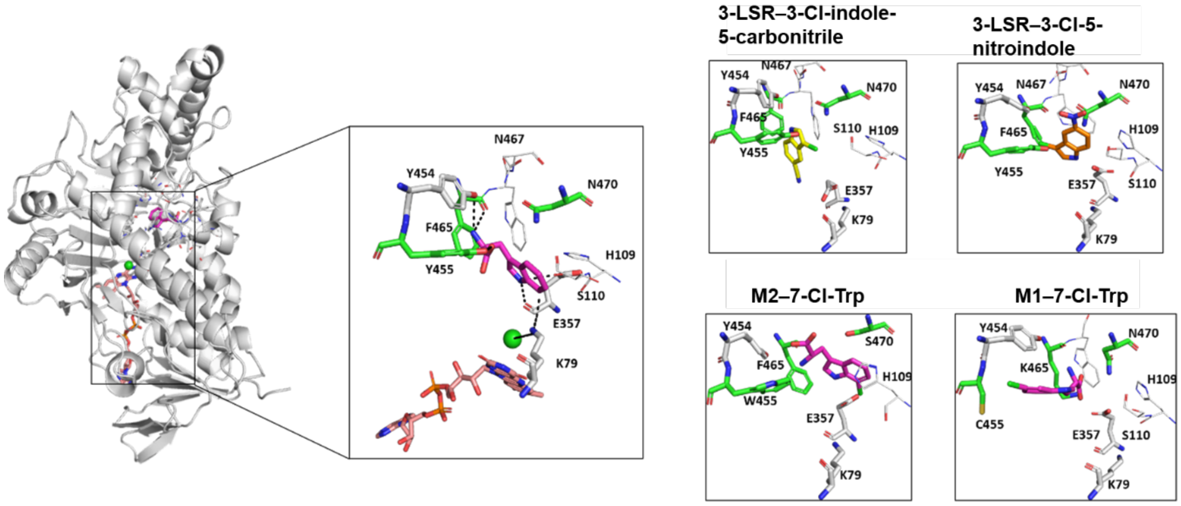

Figure S8. (left) Overall structure of 3-LSR - FAD/Cl/Trp complex. The 3-LSR is shown as grey cartoon and the bound FAD (orange sticks), Cl (green sphere), Trp (magenta sticks) are highlighted. (right) Close-up view of snapshots of 3-LSR, M1 and M2 enzymes bound to indole derivatives and Trp. For clarity only the active site residues that are interacting with the ligands are shown in sticks (grey). The residues that are mutated in the M1 and M2 are coloured (green) and the bound ligands are coloured separately. The protein–ligand interactions are highlighted in dashed lines (black).

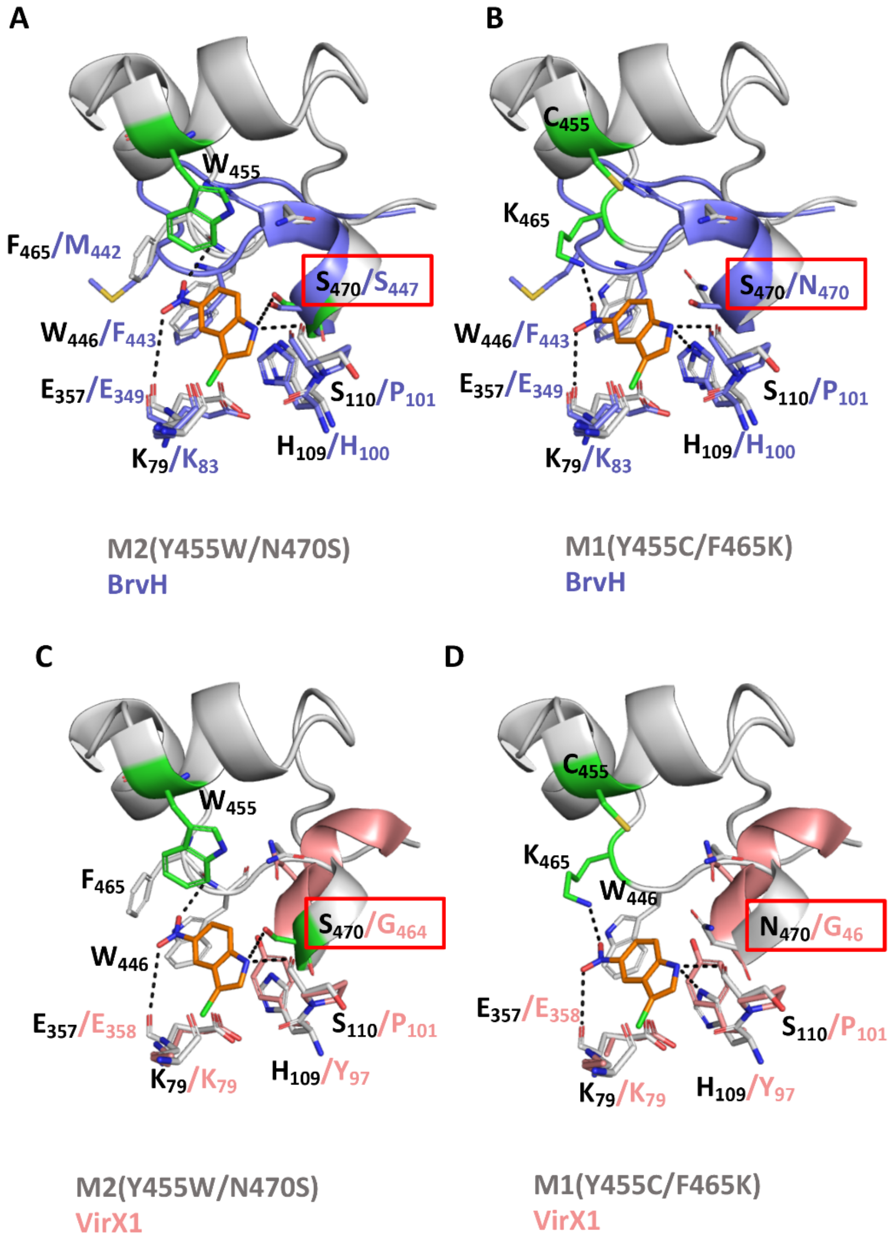

Figure S9. Structural comparison of 3-LSR M1 and M2 mutants with BrvH and VirX1. A,B) M2 and M1 (grey) structural overlay with BrvH (slate). C,D) M2 and M1 (grey) structural overlay with VirX1 (salmon). For clarity only the active site residues within the vicinity of chorinated 5-nitroindole (**2**) (orange) are shown in sticks. Residues mutated in M1 and M2 are coloured (green). The protein–ligand interactions are highlighted in dashed lines (black). Structurally equivalent residues to N470 of 3-LSR are boxed.

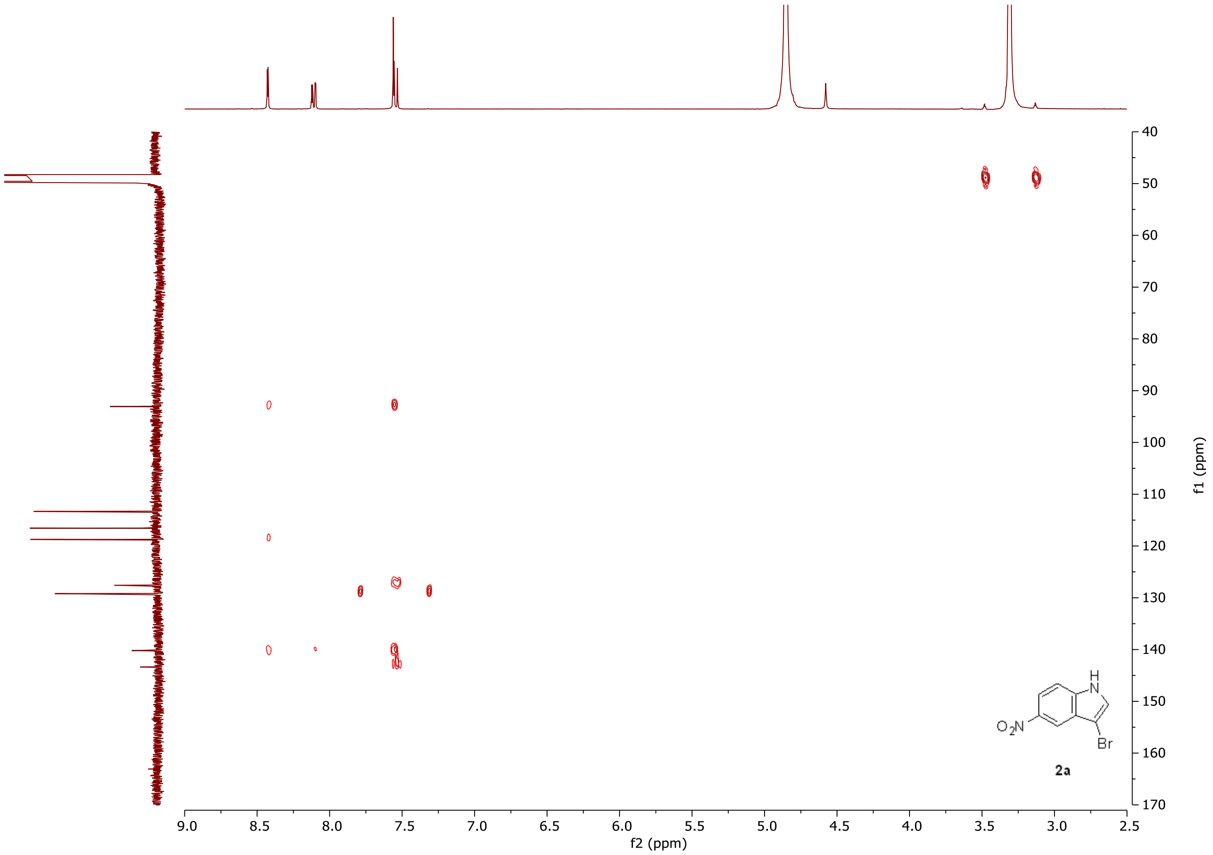

Figure S10. HMBC spectrum of 3-bromo-5-nitro-1H-indole.

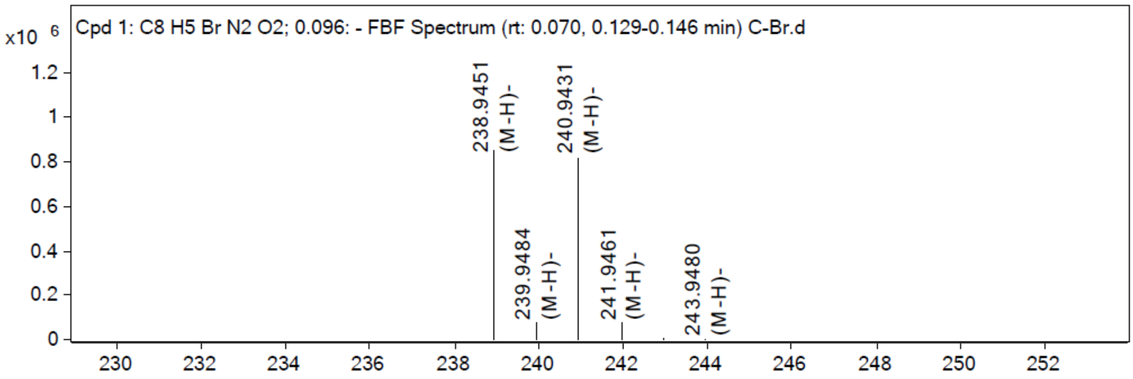

Figure S11. HRMS of 3-bromo-5-nitro-1H-indole.

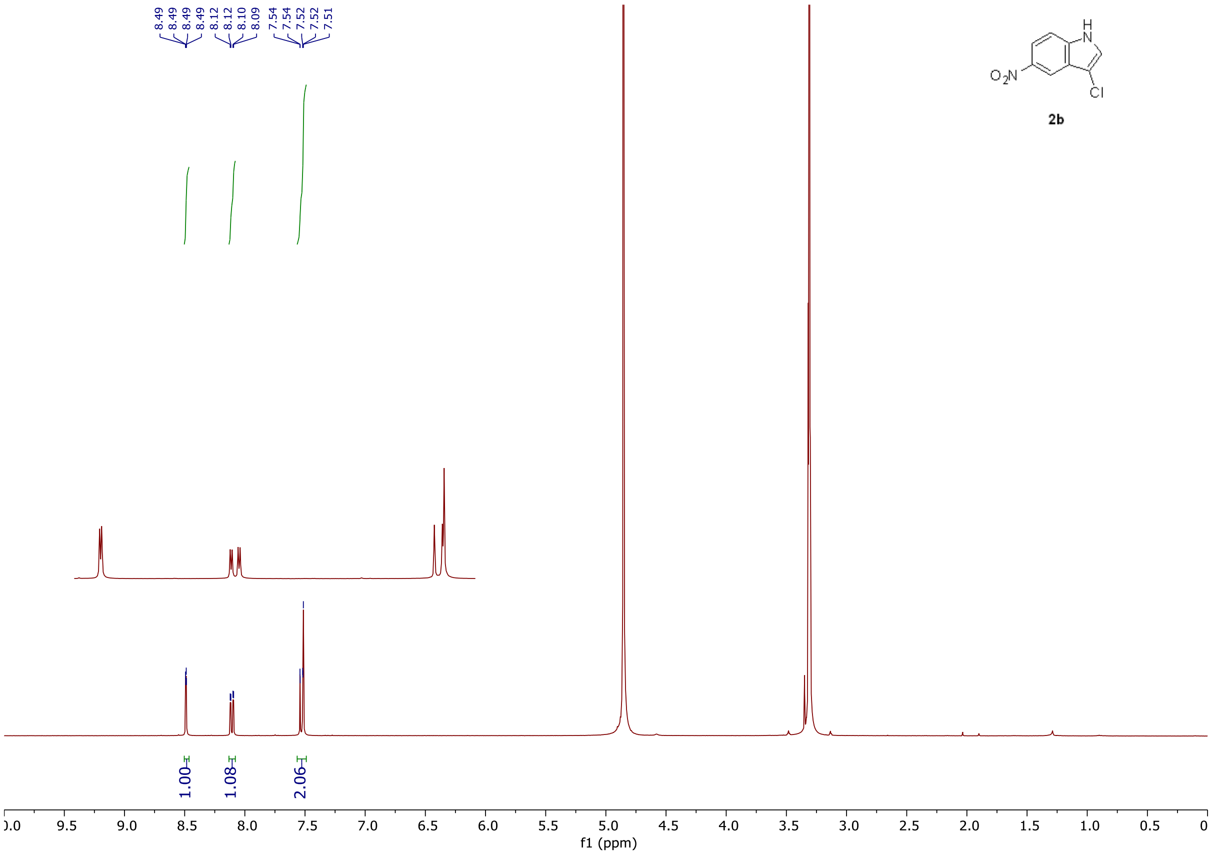

Figure S12 ^{1}H spectrum of 3-chloro-5-nitro-1H-indole

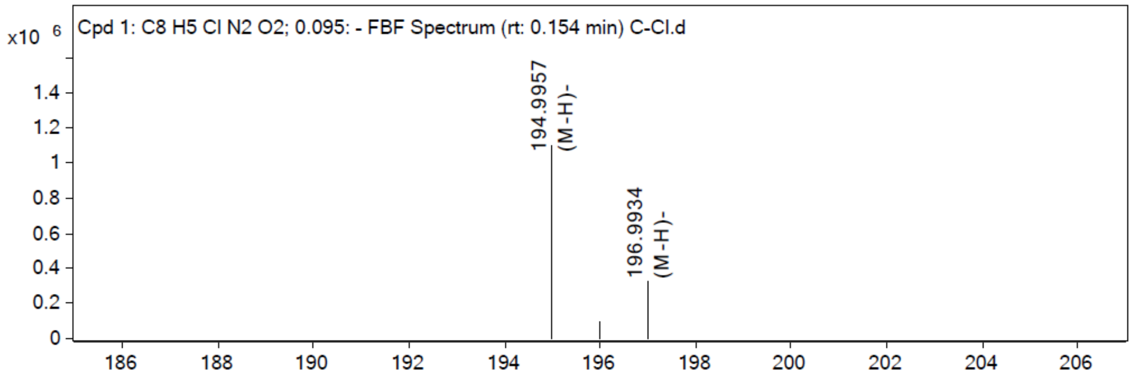

Figure S13 HRMS of 3-chloro-5-nitro-1H-indole

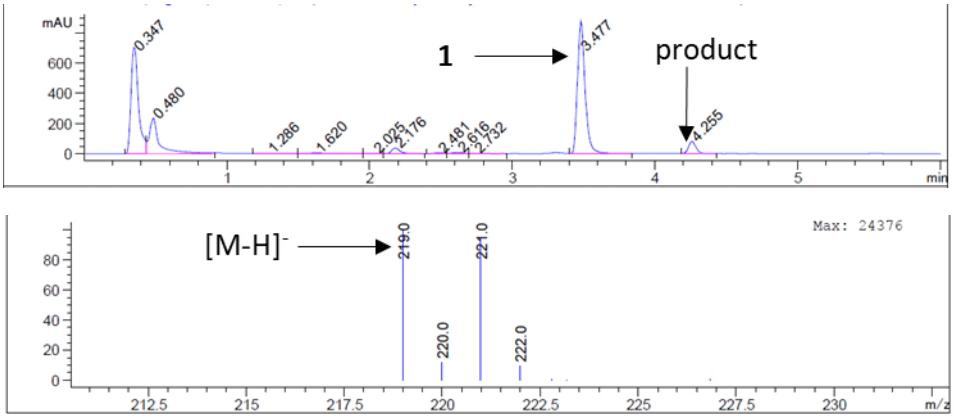

Figure S14. LC-MS spectrum of bromination of indole substrate **1** produced by enzymatic halogenation using M1. No di and tri substituted product were observed by mass extraction in MS.

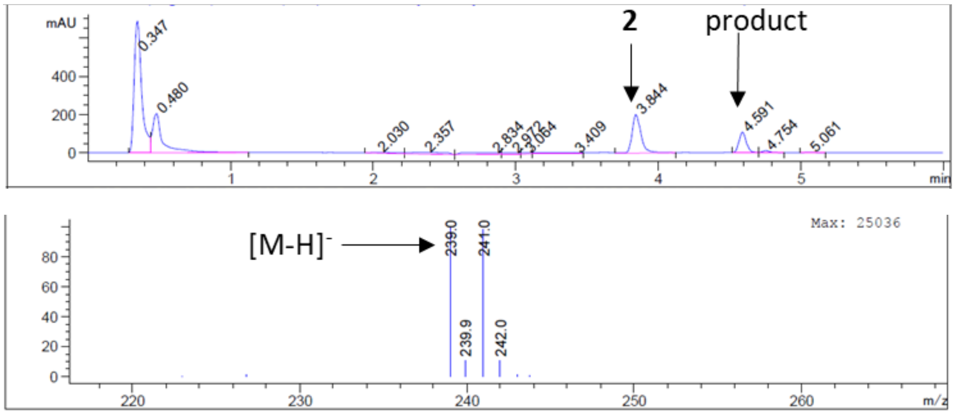

Figure S15. LC-MS spectrum of bromination of indole substrate **2** produced by enzymatic halogenation using M1. No di and tri substituted product were observed by mass extraction in MS.

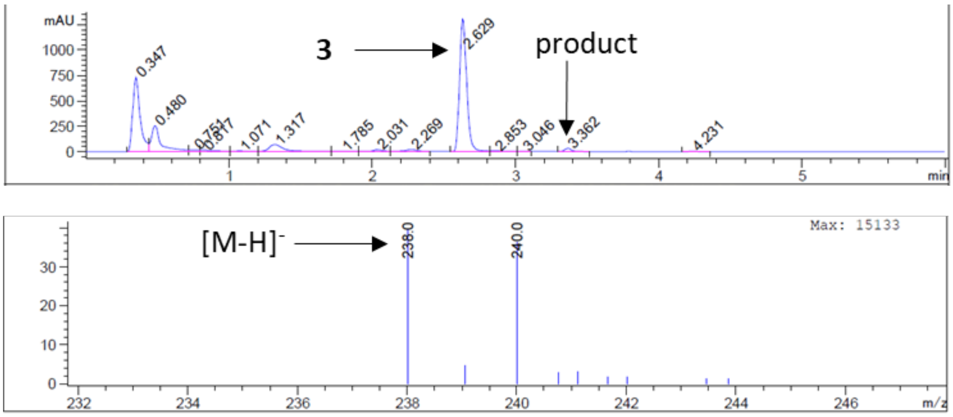

Figure S16. LC-MS spectrum of bromination of indole substrate **3** produced by enzymatic halogenation using M1. No di and tri substituted product were observed by mass extraction in MS.

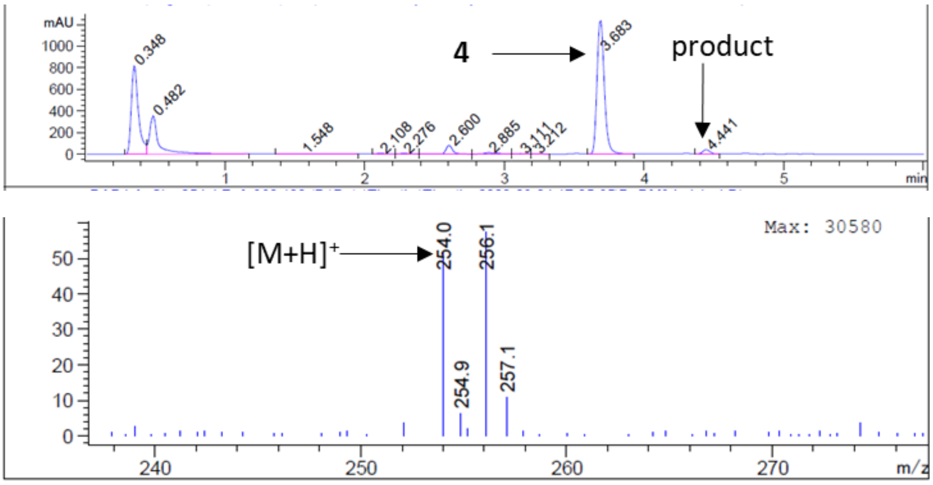

Figure S17. LC-MS spectrum of bromination of indole substrate **4** produced by enzymatic halogenation using M1. No di and tri substituted product were observed by mass extraction in MS.

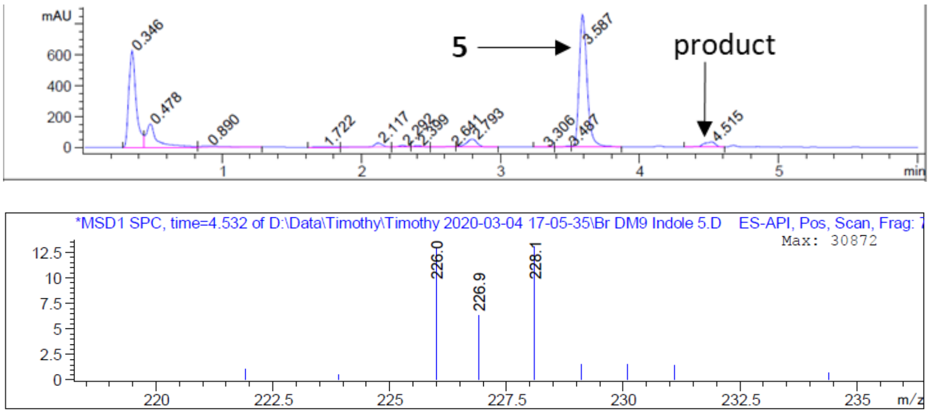

Figure S18. LC-MS spectrum of bromination of indole substrate **5** produced by enzymatic halogenation using M1. No di and tri substituted product were observed by mass extraction in MS.

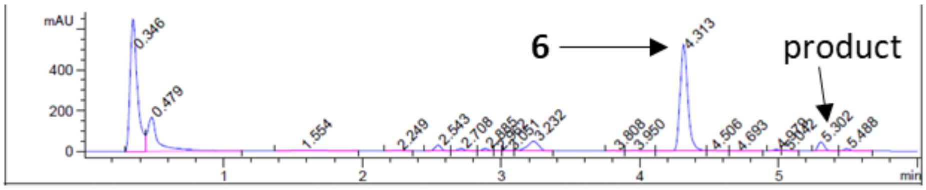

Figure S19. LC spectrum of bromination of indole substrate **6** produced by enzymatic halogenation using M1. No di and tri substituted product were observed by mass extraction in MS.

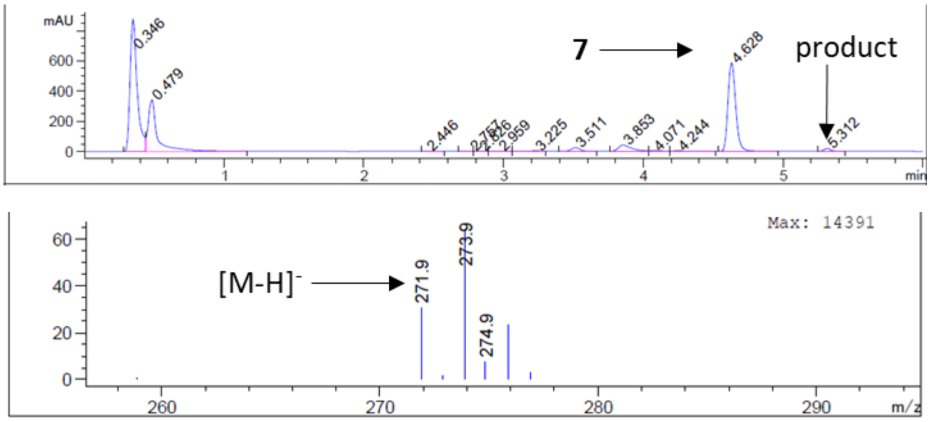

Figure S20. LC-MS spectrum of bromination of indole substrate **7** produced by enzymatic halogenation using M1. No di and tri substituted product were observed by mass extraction in MS.

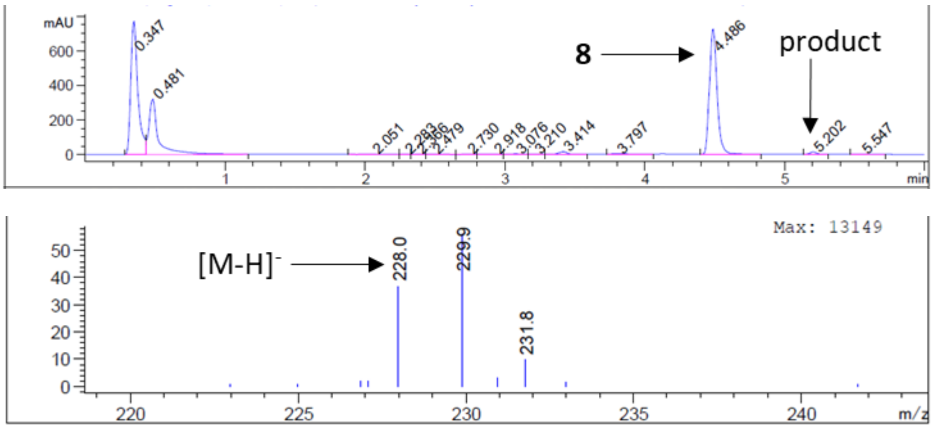

Figure S21. LC-MS spectrum of bromination of indole substrate **8** produced by enzymatic halogenation using M1. No di and tri substituted product were observed by mass extraction in MS.

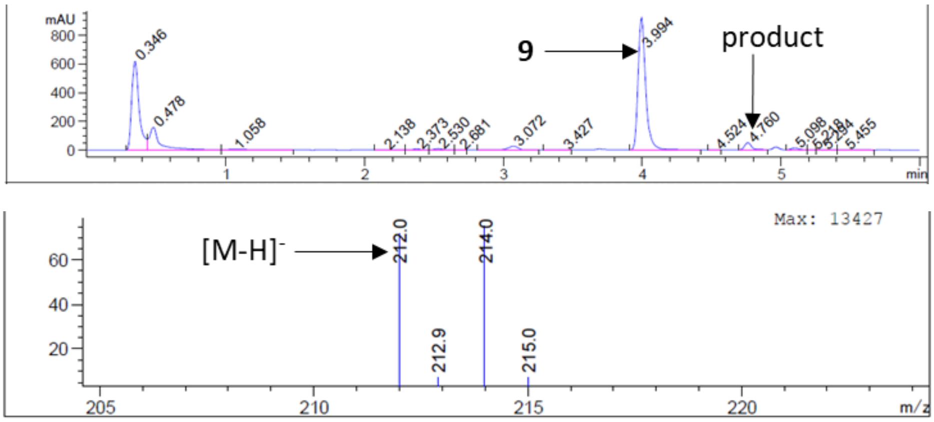

Figure S22. LC-MS spectrum of bromination of indole substrate **9** produced by enzymatic halogenation using M1. No di and tri substituted product were observed by mass extraction in MS.

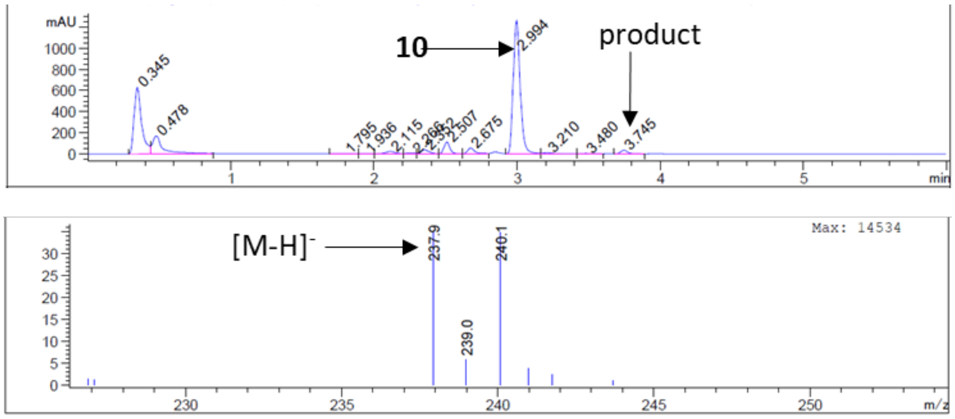

Figure S23. LC-MS spectrum of bromination of indole substrate **10** produced by enzymatic halogenation using M1. No di and tri substituted product were observed by mass extraction in MS.

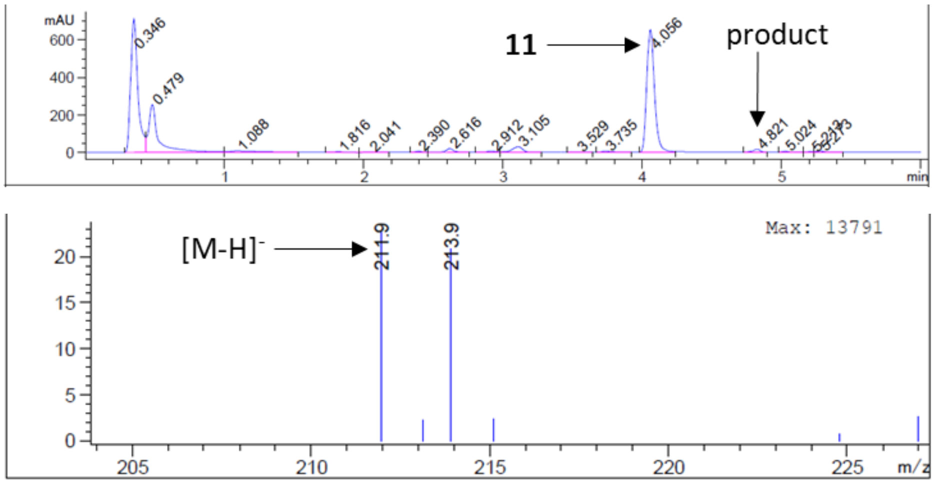

Figure S24. LC-MS spectrum of bromination of indole substrate **11** produced by enzymatic halogenation using M1. No di and tri substituted product were observed by mass extraction in MS.

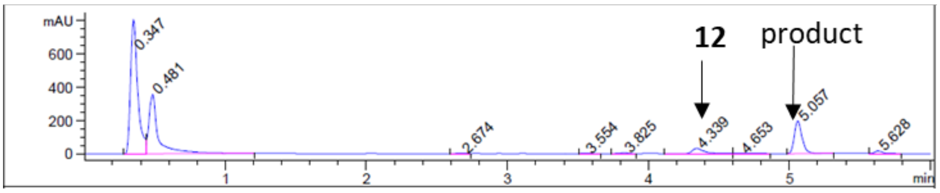

Figure S25. LC spectrum of bromination of indole substrate **12** produced by enzymatic halogenation using M1. No di and tri substituted product were observed by mass extraction in MS.

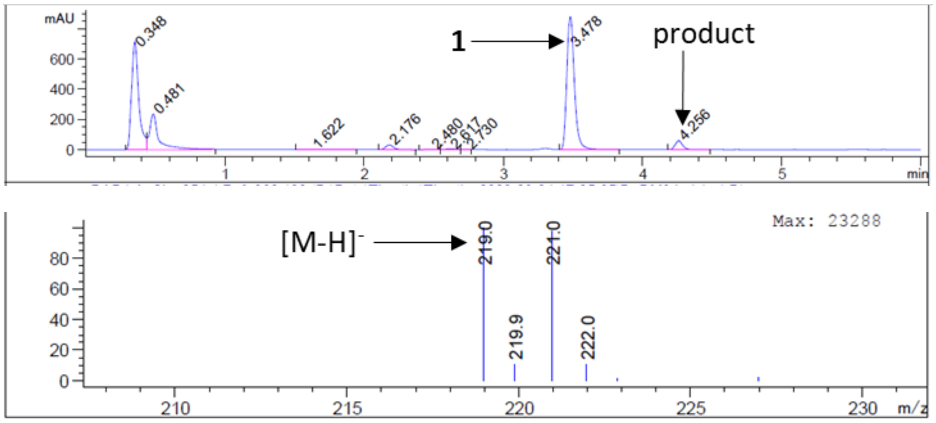

Figure S26. LC-MS spectrum of bromination of indole substrate **1** produced by enzymatic halogenation using M2. No di and tri substituted product were observed by mass extraction in MS.

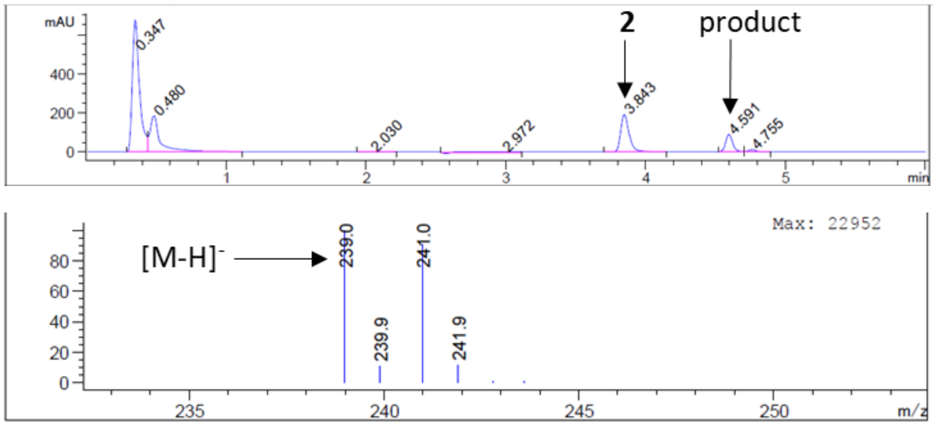

Figure S27. LC-MS spectrum of bromination of indole substrate **2** produced by enzymatic halogenation using M2. No di and tri substituted product were observed by mass extraction in MS.

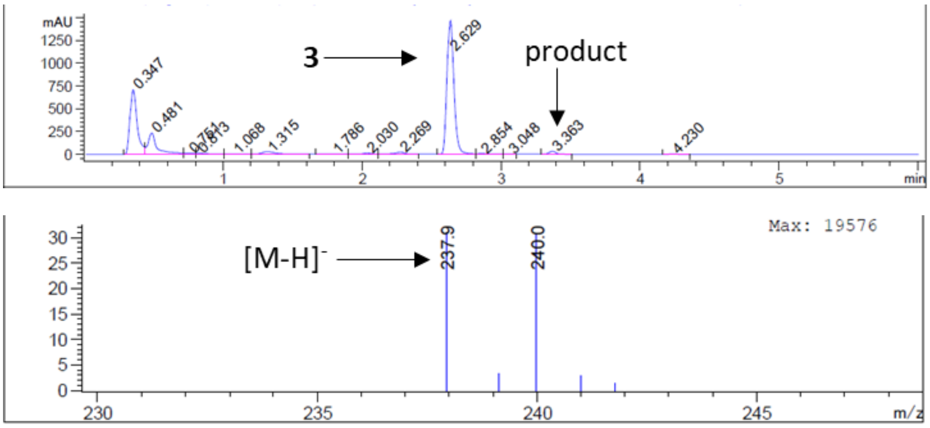

Figure S28. LC-MS spectrum of bromination of indole substrate **3** produced by enzymatic halogenation using M2. No di and tri substituted product were observed by mass extraction in MS.

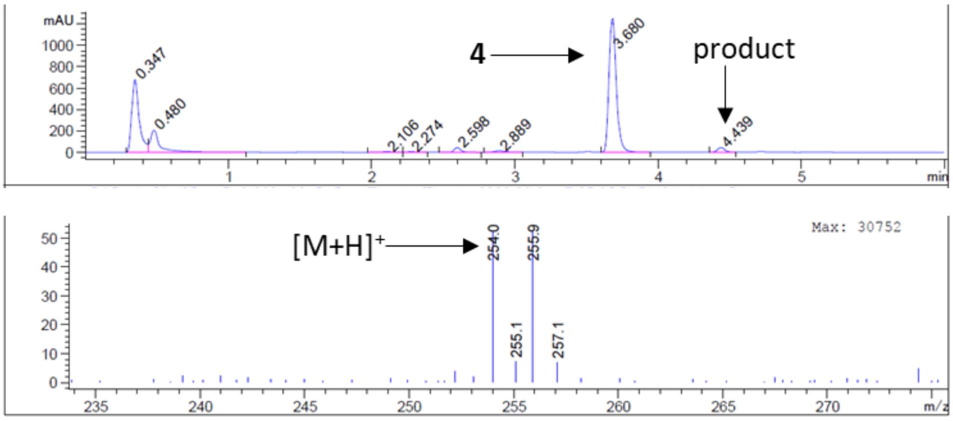

Figure S29. LC-MS spectrum of bromination of indole substrate **4** produced by enzymatic halogenation using M2. No di and tri substituted product were observed by mass extraction in MS.

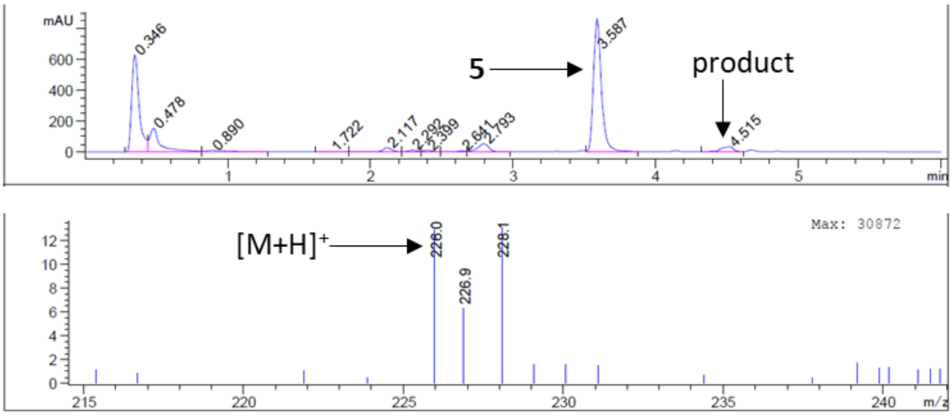

Figure S30. LC-MS spectrum of bromination of indole substrate **5** produced by enzymatic halogenation using M2. No di and tri substituted product were observed by mass extraction in MS.

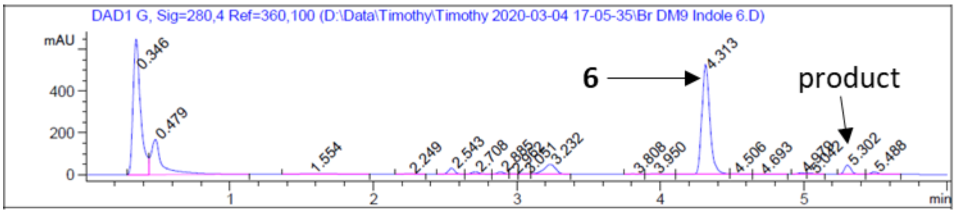

Figure S31. LC spectrum of bromination of indole substrate **6** produced by enzymatic halogenation using M2. No di and tri substituted product were observed by mass extraction in MS.

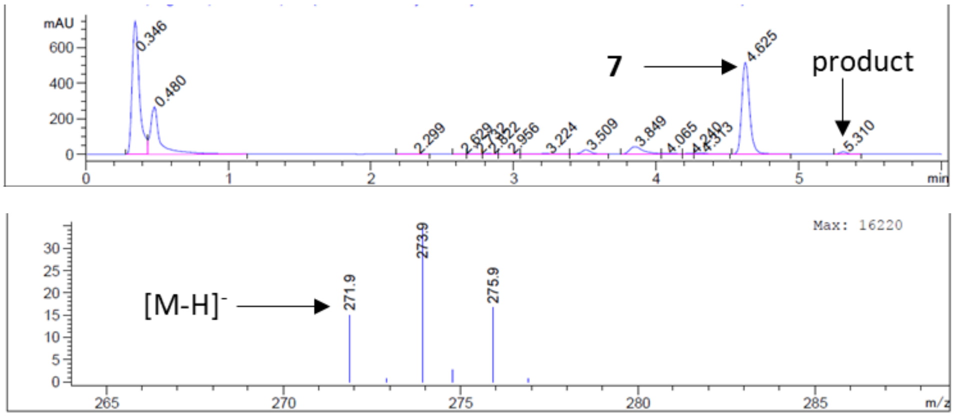

Figure S32. LC-MS spectrum of bromination of indole substrate **7** produced by enzymatic halogenation using M2. No di and tri substituted product were observed by mass extraction in MS.

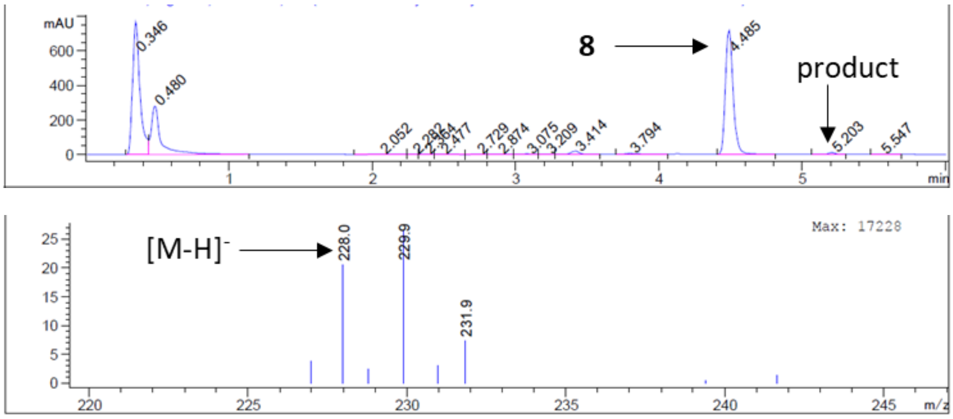

Figure S33. LC-MS spectrum of bromination of indole substrate **8** produced by enzymatic halogenation using M2. No di and tri substituted product were observed by mass extraction in MS.

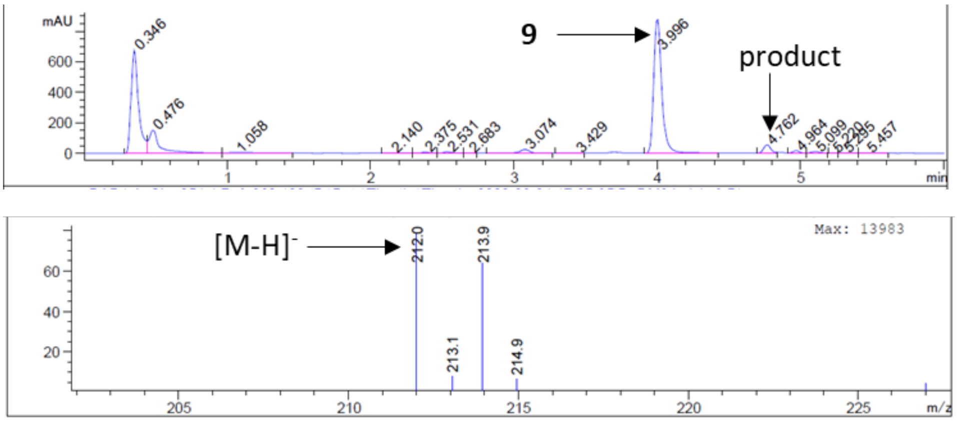

Figure S34. LC-MS spectrum of bromination of indole substrate **9** produced by enzymatic halogenation using M2. No di and tri substituted product were observed by mass extraction in MS.

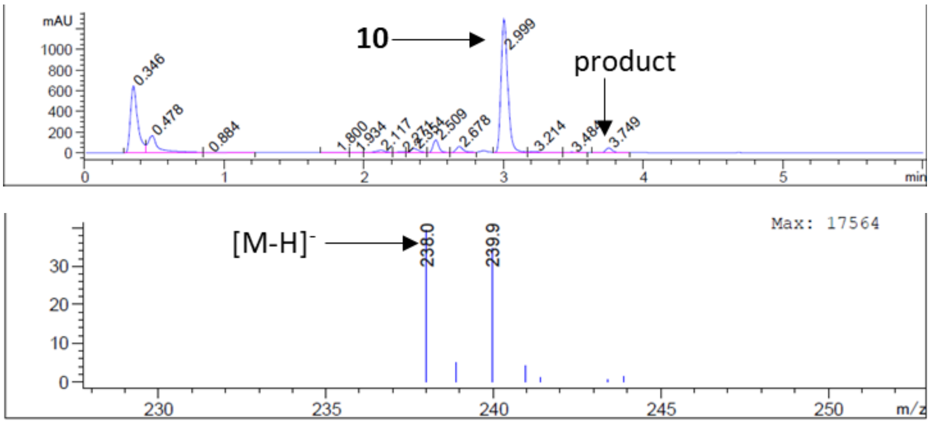

Figure S35. LC-MS spectrum of bromination of indole substrate **10** produced by enzymatic halogenation using M2. No di and tri substituted product were observed by mass extraction in MS.

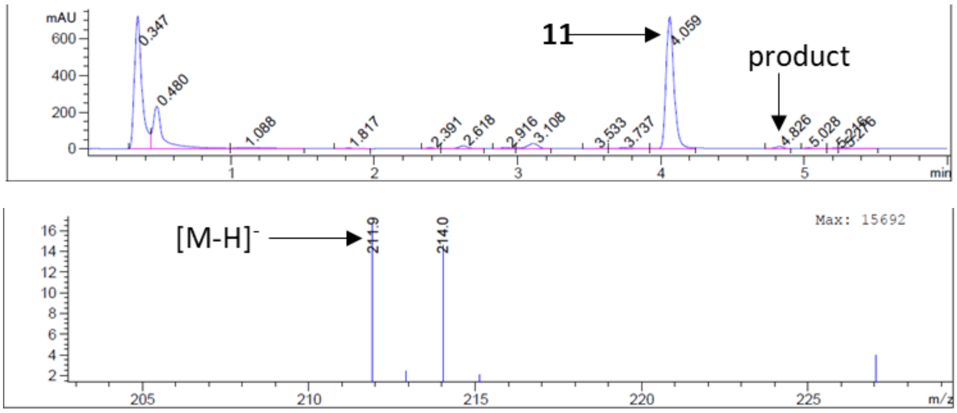

Figure S36. LC-MS spectrum of bromination of indole substrate **11** produced by enzymatic halogenation using M2. No di and tri substituted product were observed by mass extraction in MS.

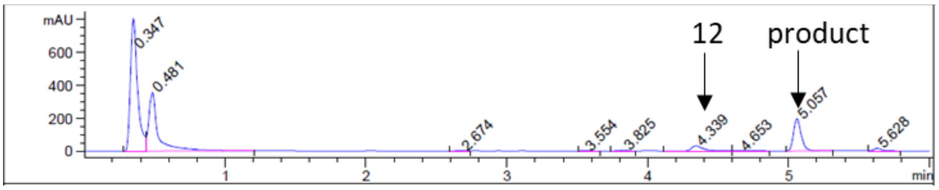

Figure S37. LC spectrum of bromination of indole substrate **12** produced by enzymatic halogenation using M2. No di and tri substituted product were observed by mass extraction in MS.

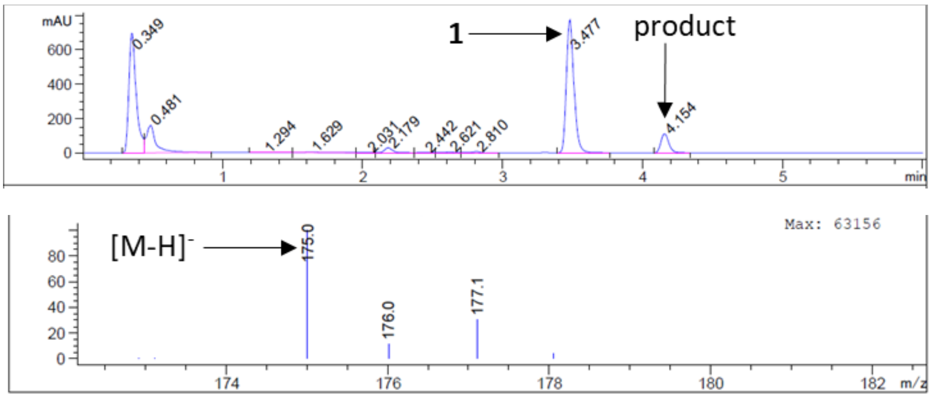

Figure S38. LC-MS spectrum of chlorination of indole substrate **1** produced by enzymatic halogenation using M1. No di and tri substituted product were observed by mass extraction in MS.

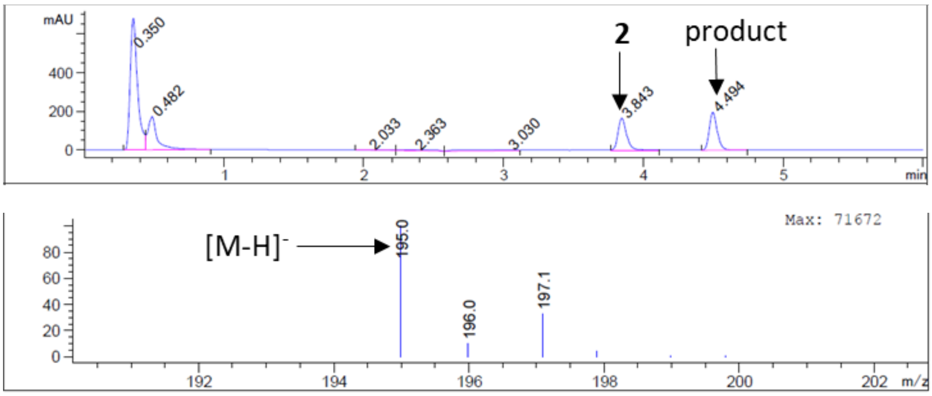

Figure S39. LC-MS spectrum of chlorination of indole substrate **2** produced by enzymatic halogenation using M1. No di and tri substituted product were observed by mass extraction in MS.

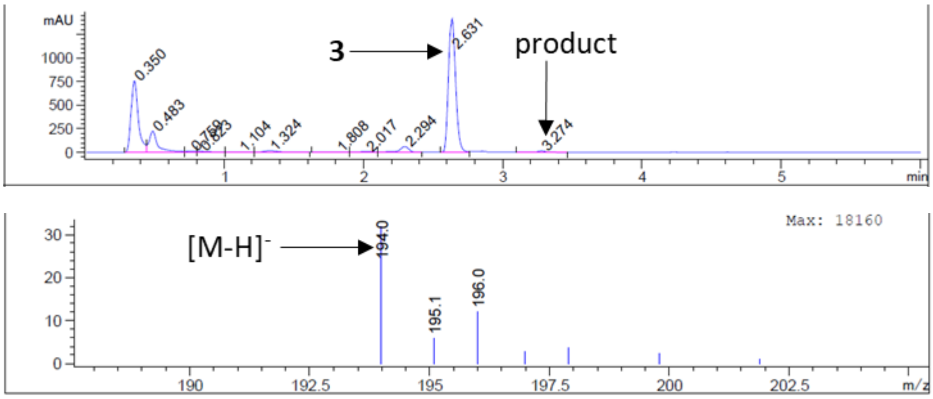

Figure S40. LC-MS spectrum of chlorination of indole substrate **3** produced by enzymatic halogenation using M1. No di and tri substituted product were observed by mass extraction in MS.

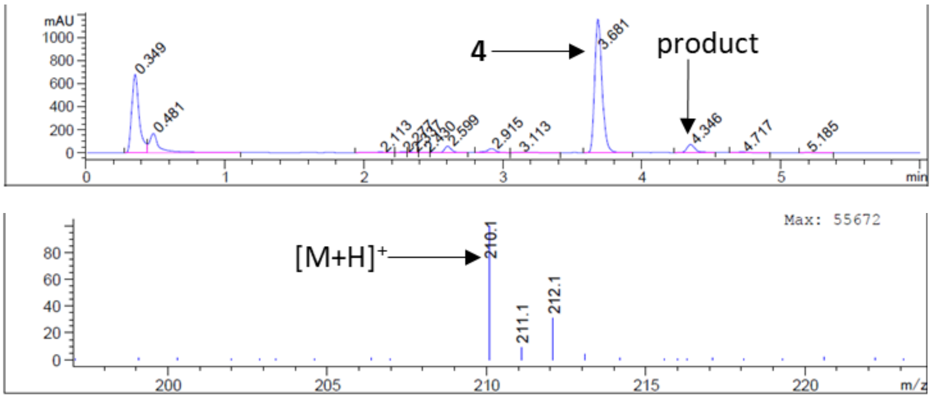

Figure S41. LC-MS spectrum of chlorination of indole substrate **4** produced by enzymatic halogenation using M1. No di and tri substituted product were observed by mass extraction in MS.

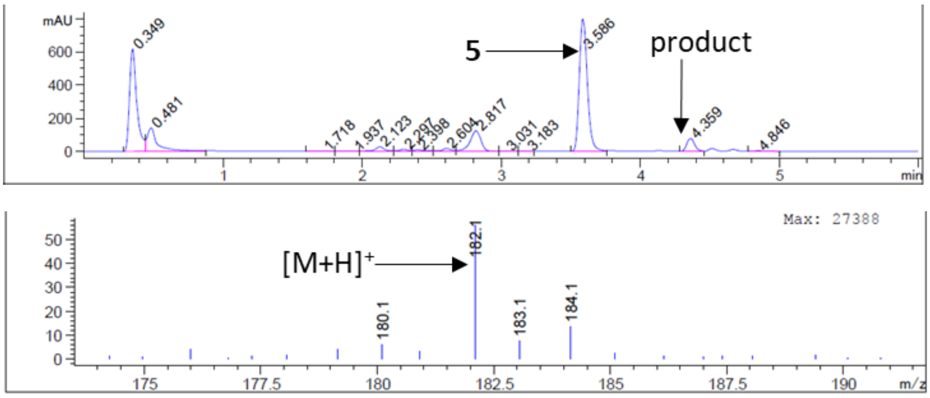

Figure S42. LC-MS spectrum of chlorination of indole substrate **5** produced by enzymatic halogenation using M1. No di and tri substituted product were observed by mass extraction in MS.

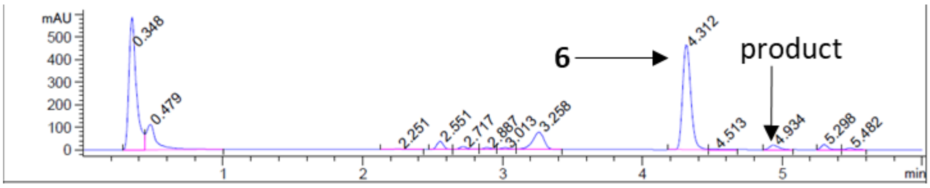

Figure S43. LC spectrum of chlorination of indole substrate **6** produced by enzymatic halogenation using M1. No di and tri substituted product were observed by mass extraction in MS.

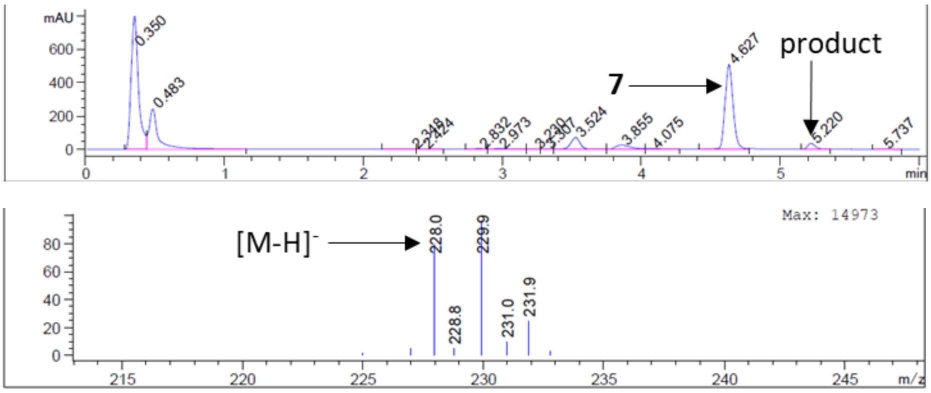

Figure S44. LC-MS spectrum of chlorination of indole substrate **7** produced by enzymatic halogenation using M1. No di and tri substituted product were observed by mass extraction in MS.

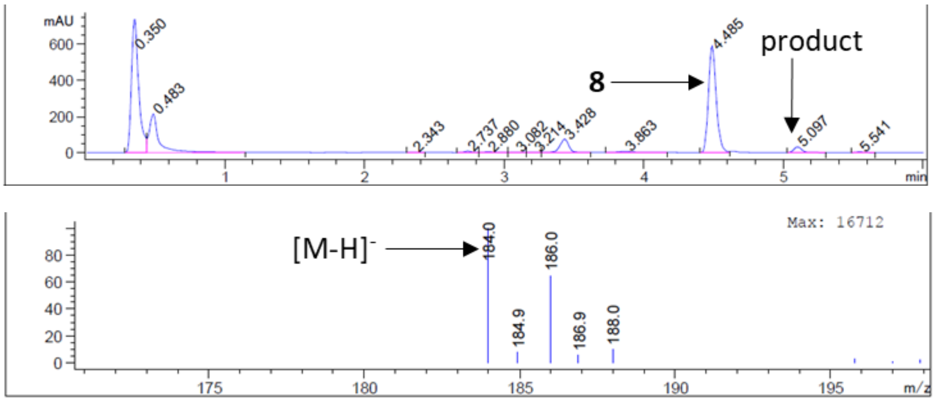

Figure S45. LC-MS spectrum of chlorination of indole substrate **8** produced by enzymatic halogenation using M1. No di and tri substituted product were observed by mass extraction in MS.

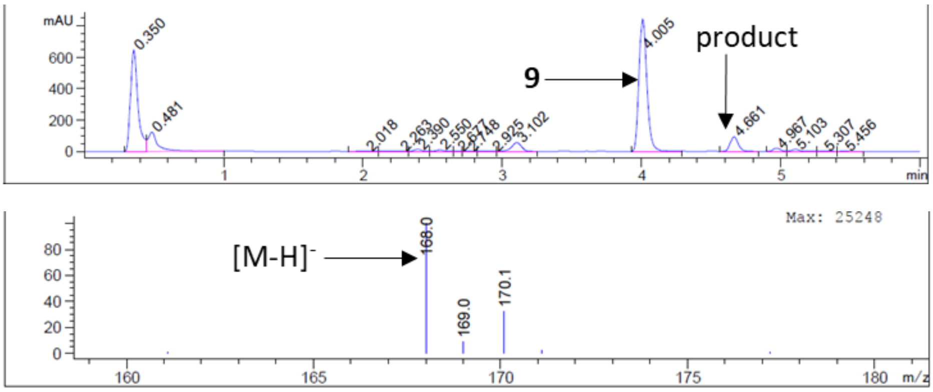

Figure S46. LC-MS spectrum of chlorination of indole substrate **9** produced by enzymatic halogenation using M1. No di and tri substituted product were observed by mass extraction in MS.

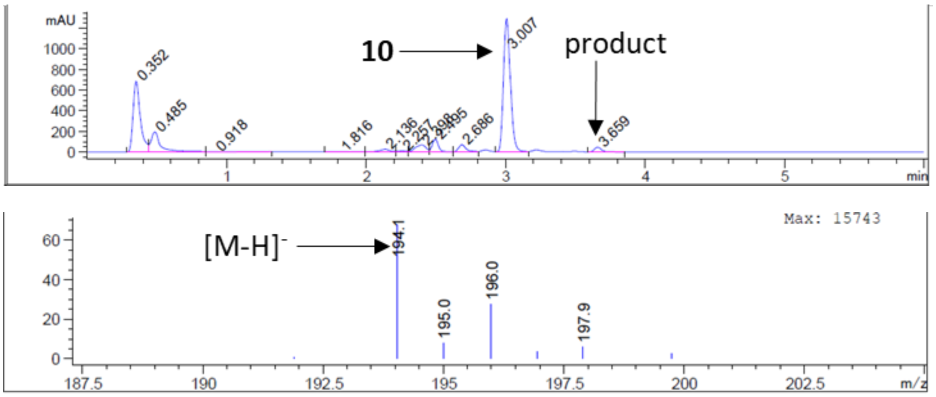

Figure S47. LC-MS spectrum of chlorination of indole substrate **10** produced by enzymatic halogenation using M1. No di and tri substituted product were observed by mass extraction in MS.

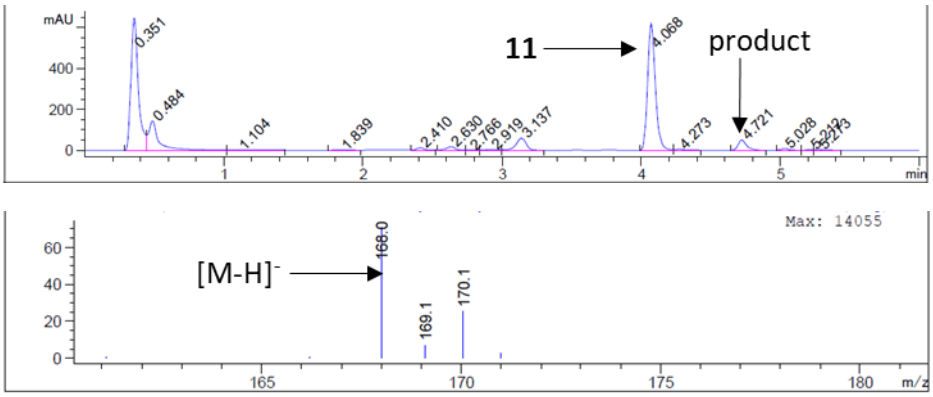

Figure S48. LC-MS spectrum of chlorination of indole substrate **11** produced by enzymatic halogenation using M1. No di and tri substituted product were observed by mass extraction in MS.

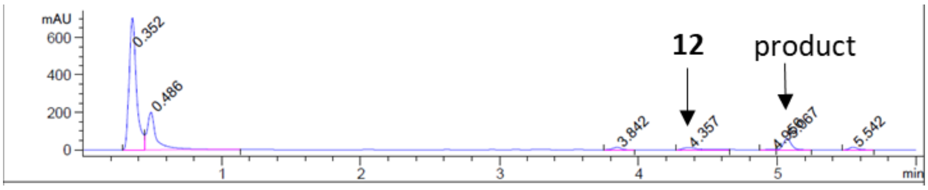

Figure S49. LC spectrum of chlorination of indole substrate **12** produced by enzymatic halogenation using M1. No di and tri substituted product were observed by mass extraction in MS.

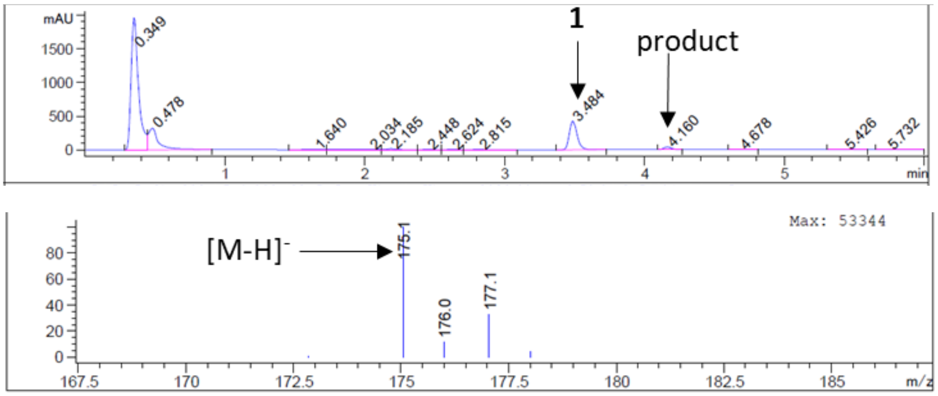

Figure S50. LC-MS spectrum of chlorination of indole substrate **1** produced by enzymatic halogenation using M2. No di and tri substituted product were observed by mass extraction in MS.

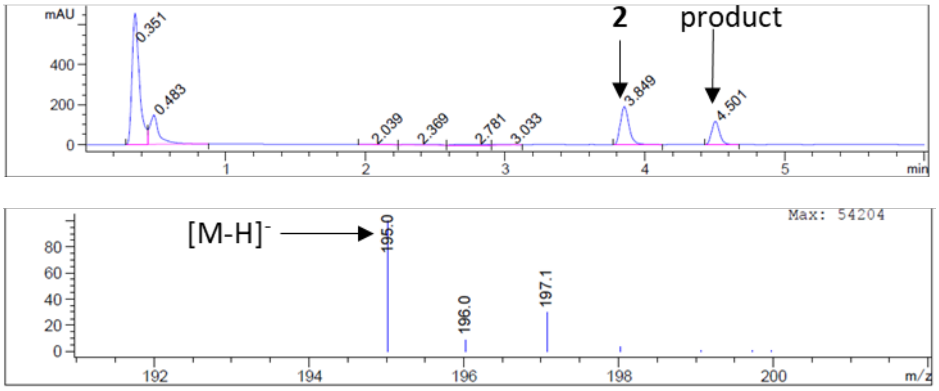

Figure S51. LC-MS spectrum of chlorination of indole substrate **2** produced by enzymatic halogenation using M2. No di and tri substituted product were observed by mass extraction in MS.

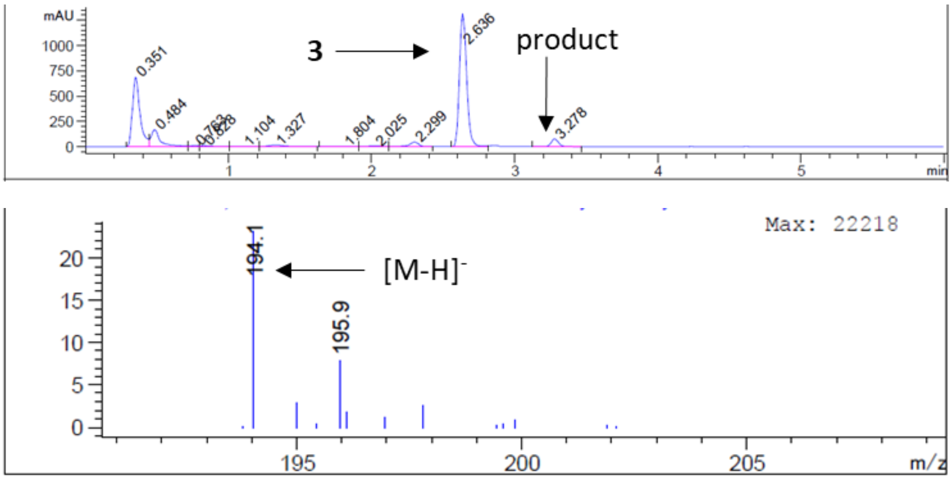

Figure S52. LC-MS spectrum of chlorination of indole substrate **3** produced by enzymatic halogenation using M2. No di and tri substituted product were observed by mass extraction in MS.

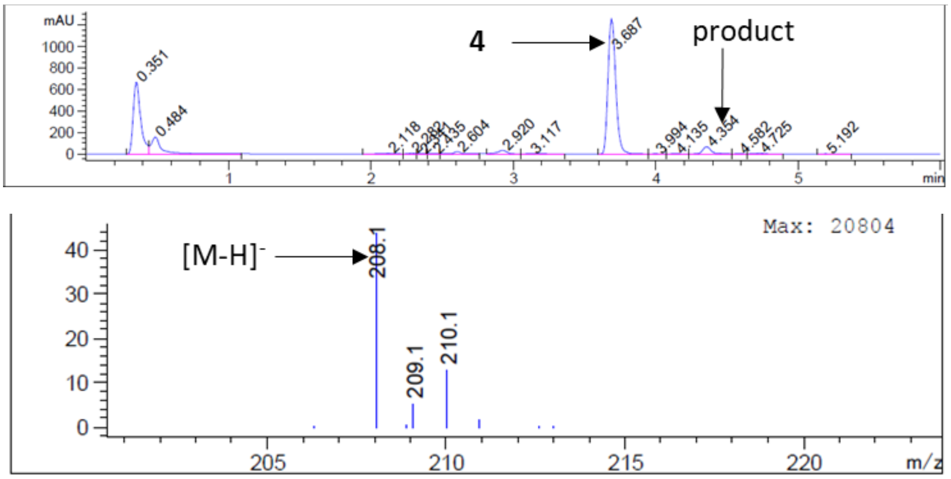

Figure S53. LC-MS spectrum of chlorination of indole substrate **4** produced by enzymatic halogenation using M2. No di and tri substituted product were observed by mass extraction in MS.

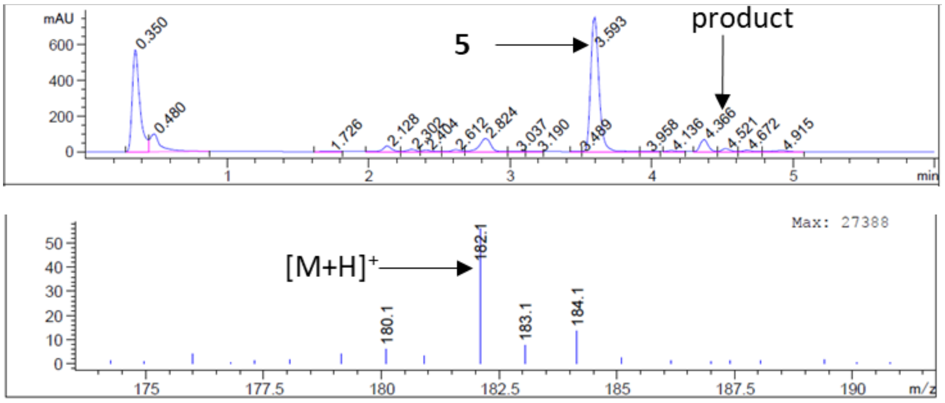

Figure S54. LC-MS spectrum of chlorination of indole substrate **5** produced by enzymatic halogenation using M2. No di and tri substituted product were observed by mass extraction in MS.

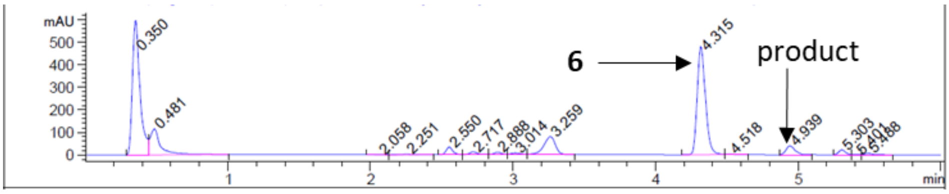

Figure S55. LC spectrum of chlorination of indole substrate **6** produced by enzymatic halogenation using M2. No di and tri substituted product were observed by mass extraction in MS.

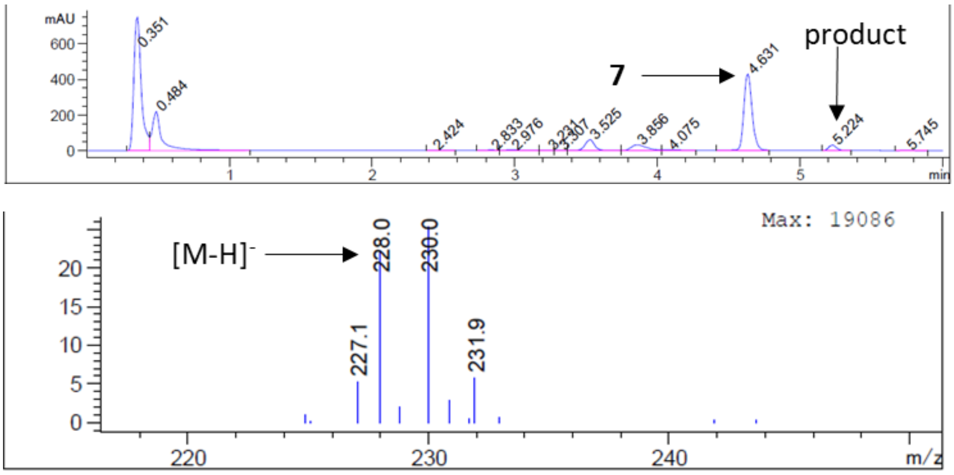

Figure S56. LC-MS spectrum of chlorination of indole substrate **7** produced by enzymatic halogenation using M2. No di and tri substituted product were observed by mass extraction in MS.

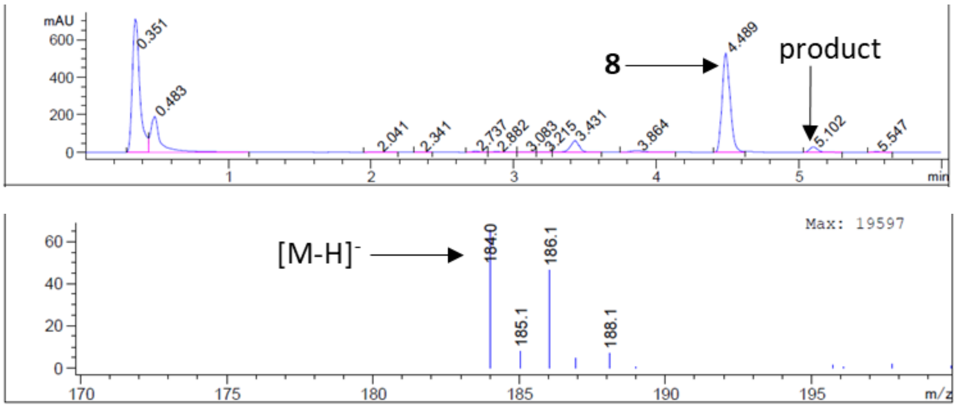

Figure S57. LC-MS spectrum of chlorination of indole substrate **8** produced by enzymatic halogenation using M2. No di and tri substituted product were observed by mass extraction in MS.

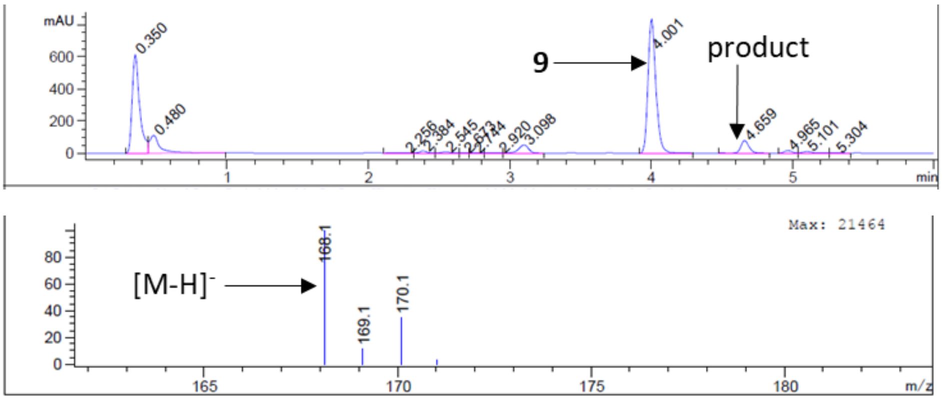

Figure S58. LC-MS spectrum of chlorination of indole substrate **9** produced by enzymatic halogenation using M2. No di and tri substituted product were observed by mass extraction in MS.

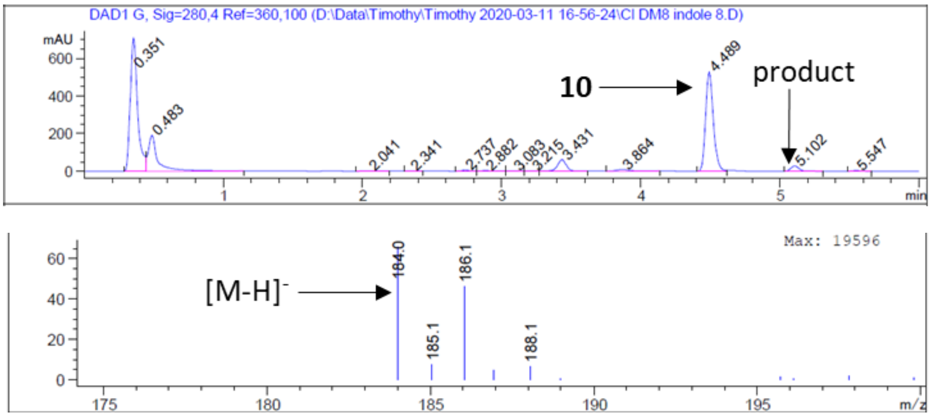

Figure S59. LC-MS spectrum of chlorination of indole substrate **10** produced by enzymatic halogenation using M2. No di and tri substituted product were observed by mass extraction in MS.

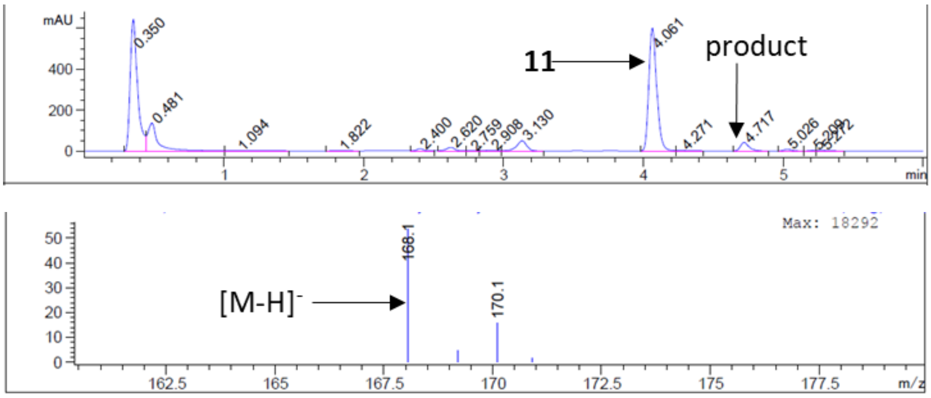

Figure S60. LC-MS spectrum of chlorination of indole substrate **11** produced by enzymatic halogenation using M2. No di and tri substituted product were observed by mass extraction in MS.

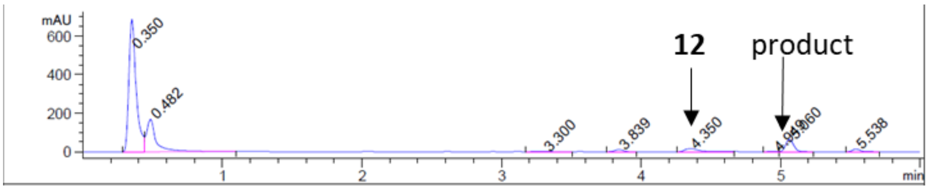

Figure S61. LC spectrum of chlorination of indole substrate **12** produced by enzymatic halogenation using M2. No di and tri substituted product were observed by mass extraction in MS.

Supplementary data

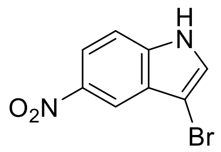

3-bromo-5-nitro-1H-indole (2a)

^{1}H NMR (400 MHz, CD_3OD): δ 8.43 (d, *J* = 2.2 Hz, 1H), 8.11 (dd, *J* = 9.0, 2.3 Hz, 1H), 7.59 – 7.51 (m, 2H). ^{13}C NMR (101 MHz, CD_3OD): 143.4, 140.2, 129.2, 127.6, 118.7, 116.5, 113.3, 93.1. HRMS (ESI, M-) m/z calculated for $C_8H_5{}^{79}BrN_2O_2$ 238.9461, found 238.9451.

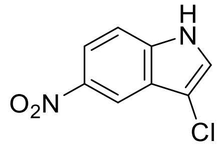

3-chloro-5-nitro-1H-indole (2b)

^{1}H NMR (400 MHz, CD_3OD): 8.49 (dd, *J* = 2.3, 0.5 Hz, 1H), 8.11 (dd, *J* = 9.0, 2.3 Hz, 1H), 7.56 – 7.48 (m, 2H). HRMS (ESI, M-) m/z calculated for $C_8H_5ClN_2O_2$ 194.9967, found 194.9957.
